# Shape controlled synthesis of porous tetrametallic PtAgBiCo nanoplates as highly active and methanol-tolerant electrocatalyst for oxygen reduction reaction†
†Electronic supplementary information (ESI) available. See DOI: 10.1039/c7sc00318h



**DOI:** 10.1039/c7sc00318h

**Published:** 2017-03-22

**Authors:** Azhar Mahmood, Nanhong Xie, Muhammad Aizaz Ud Din, Faisal Saleem, Haifeng Lin, Xun Wang

**Affiliations:** a Key Lab of Organic Optoelectronics and Molecular Engineering , Department of Chemistry , Tsinghua University , Beijing , 100084 , China . Email: wangxun@mail.tsinghua.edu.cn

## Abstract

Mechanistic control is a powerful means for manufacturing specific shapes of metal nanostructures and optimizing their performance in a variety of applications. Thus, we successfully synthesized multimetallic nanoplates (PtAgBiCo and PtAgBi) by combining the concepts of crystal symmetry, oxidative etching and seed ratio, and tuned their activity, stability and methanol tolerance, as well as Pt utilization, for the oxygen reduction reaction in direct methanol fuel cells. Systematic studies reveal that the formation of PtAgBiCo triangular nanoplates with a high morphological yield (>90%) can be achieved by crystallinity alteration, while electrochemical measurements indicate that the PtAgBiCo nanoplates have superior electrocatalytic activity towards the oxygen reduction reaction. The specific and mass activity of the PtAgBiCo nanoplates are 8 and 5 times greater than that of the commercial Pt/C catalyst, respectively. In addition, the tetrametallic PtAgBiCo nanoplates exhibit a more positive half-wave potential for the oxygen reduction reaction and possess an excellent methanol tolerance limit compared with the commercial Pt/C catalyst.

## Abstract 2

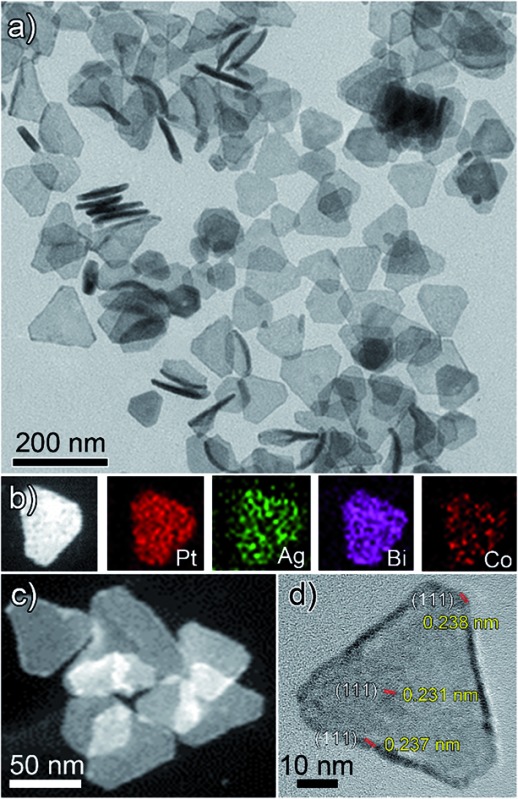
We present a new two-step synthetic route combining the concepts of crystal symmetry, seed ratio and oxidative etching that yields tetrametallic PtAgBiCo nanoplates.

## Introduction

The synthesis of multimetallic noble metal (Ag, Au, Pt, Pd) based nanocrystals has been recognized as a research theme due to their novel nanostructures and broad applications.^1^ Among the various shapes of noble metal based materials (wires, sheets and tetrapods),^2,3^ one of the most interesting shapes is nanoplates^4^ (NPLs). Their morphology plays a very important role in the determination of their properties, and they can be used as electrocatalysts in a direct methanol fuel cell^5^ (DMFC). Considering their synthesis and application, problems with noble metal based nanostructures include the presence of impurities with less size control in production *e.g.*, “nanoplates” and their reduction in efficiency due to crossover of methanol from the anode to the cathode through the electrolyte membrane and the poisoning effect by carbon monoxide (CO).^6,7^ After great efforts, researchers have been able to solve these challenges and found out that size controlled NPLs can be achieved in high yield using photo-induced,^4,6^ seed-mediated,^8,9^ “magic” reagent,^10^ oxidative etching,^11,12^ seed ratio^13^ and reduction rate^14^ techniques, as well as using the crystal symmetry of the starting nuclei.^15–17^ To enhance the catalyst durability, designed catalysts should possess both good methanol tolerance and optimal activity for the oxygen reduction reaction (ORR).^18,19^ Plate-like structures are more often observed in limited noble metals (Ag, Au and Pd)^4,6,11^ and researchers have found that the internal structure of NPLs is indeed very complex and it is challenging to equalize the size of plate-like structures.^10^ It has been proposed that the reason for such low yields of NPLs is due to the poor control over the crystal habits of the seeds, which ultimately determines the crystallinity and shape of the resulting nanoplates.^6,11^ Kelly and Sigmund *et al.* explained in detail the formation mechanism of silver and gold NPLs on the basis of twins.^16,17^ Furthermore, Xia and co-workers pointed out the overall formation mechanism of NPLs by interweaving both surface chemistry and crystallographic arguments.^14^ From the viewpoint of catalysis, to achieve both a high mass activity and methanol tolerance, doping Pt with a transition or main group metal might be an effective strategy, because the incorporation of the other metals into Pt-based alloys leads to a modification of the Pt electronic structure, especially transition metals (Mn, Co, Fe, and Ni) that easily transfer electrons to Pt which causes lattice contraction. Thus, the Pt–Pt distance becomes more favourable and the catalytic activity of Pt is improved.^20^ Previously, researchers have proved that ternary^21^ alloys show higher catalytic performance than binary alloys^22^ for the ORR. Furthermore, our group has recently proved that Pt-based tetrametallic nanosheets exhibit higher catalytic performance and methanol tolerance.^19^ Considering that Bi has been utilized to improve the methanol tolerance of Pt catalysts, co-doping Pt with Bi and one or two other metals seems to be a feasible method to design promising ORR catalysts.^19^ There are many approaches to synthesizing noble metal based plate-like structures.^4,6,8–17^ But despite incredible progress, interesting advances, and continuously improving understanding of synthesis, the challenge of systematic shape controlled synthesis of multimetallic plate-like structures has been met with limited success, because of factors like complex reaction steps, low yields, unsatisfactory uniformity and a low degree of alloying. It still remains a great challenge to form a high yield of shape controlled multimetallic NPLs.^23^ Notably, to date, no one has reported tetrametallic Pt-based triangular nanoplates.

## Results and discussion

Herein, for the first time, we present a new two-step synthetic route combining the concepts of crystal symmetry,^16,17^ seed ratio^13^ and oxidative etching^11,12^ that yields tetrametallic PtAgBiCo nanoplates. In the first step we prepared a gel like material, which was used as a template for the growth and formation of multimetallic NPLs in the second step. The structure of the NPLs was characterized by transmission electron microscopy (TEM), high-angle annular dark field scanning TEM (HAADF-STEM), high resolution TEM (HRTEM), and energy dispersive X-ray (EDX) mapping images, which confirm the formation of NPLs under the given conditions.

**Fig. 1:**
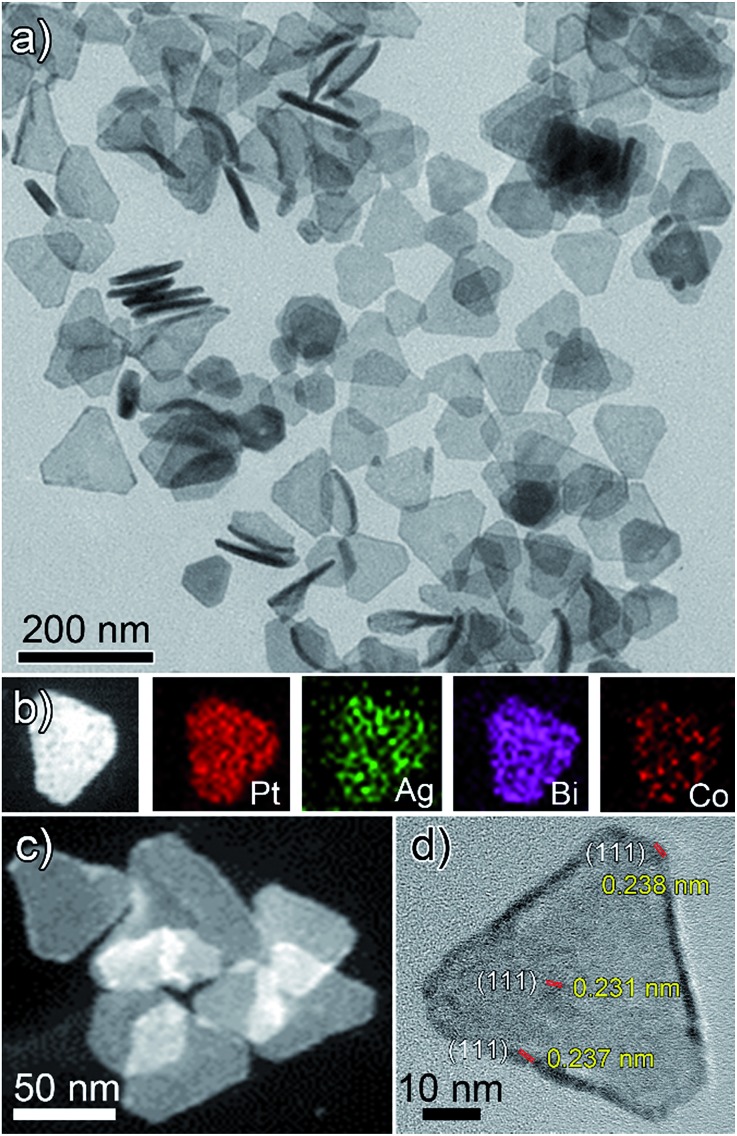
(a) TEM, (b and c) HAADF-STEM and corresponding EDX mapping; (d) HRTEM images of PtAgBiCo nanoplates.

**Fig. 2:**
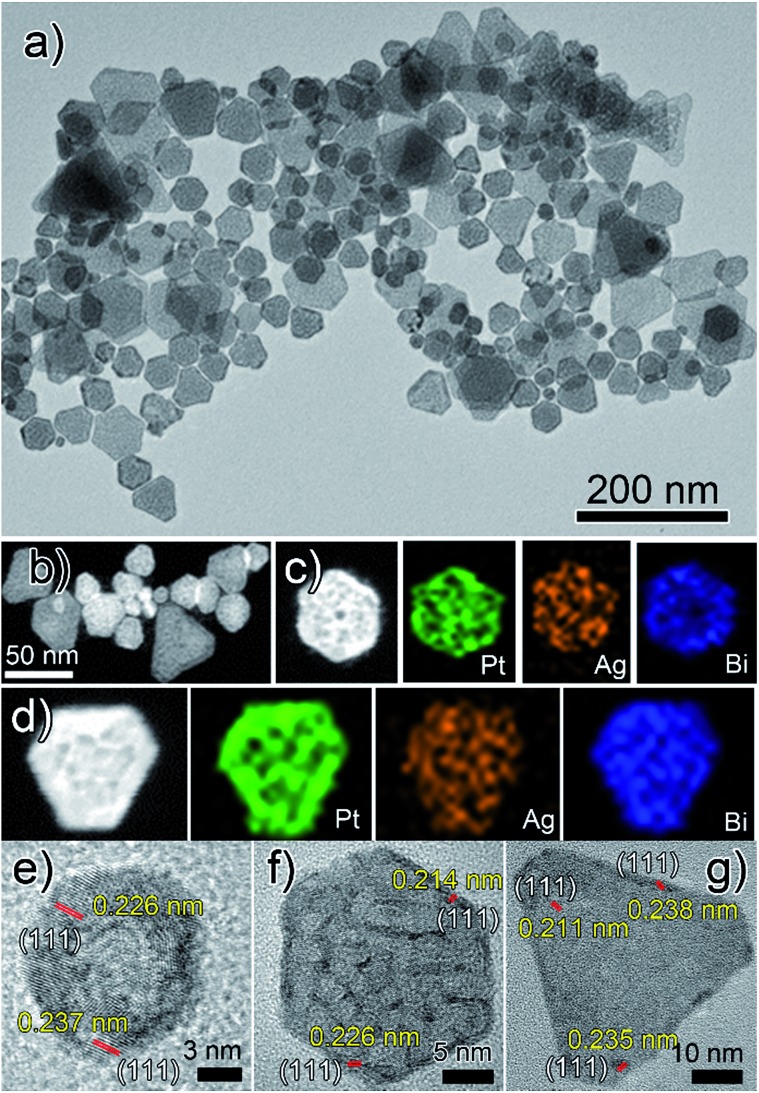
(a) TEM, (b–d) HAADF-STEM and corresponding EDX mapping; (e and f) hexagonal and (g) triangular HRTEM images of PtAgBi nanoplates.

As is shown in Fig. 1a, PtAgBiCo triangular NPLs have been successfully synthesized. From TEM characterization, it is determined that the morphological yield of PtAgBiCo triangular NPLs is over 90%, and of PtAgBi NPLs obtained by harnessing crystallinity alteration and seed ratio shown in Fig. 2a. The HRTEM image taken for tetrametallic triangular NPLs shows a lattice spacing of 0.231 nm which matches well with the calculated value of {111}-planes (Fig. 1d and S1†). For the trimetallic NPLs, it is clearly found that the product consists of two kind of morphologies (hexagonal and triangular) with varying edge sizes (13, 34 and 43 nm, Fig. 2e–g). The HRTEM images taken for hexagonal NPLs show lattice spacings of 0.226 nm and 0.214 nm (Fig. 2e and f, S2 and S3†) respectively, while the image taken for trimetallic triangular NPLs shows a lattice spacing of 0.235 nm Fig. 2g and S4† which matches well with the calculated value of {111}-planes. To get further structural information about the multimetallic NPLs, X-ray diffraction (XRD) analysis was performed (Fig. S5†). We observed that the structure (shape and edge lengths) of the NPLs is dependent on the amount of the added cobalt. This dependence of the NPLs shapes and edge lengths (Fig. S6†) on the molar concentration of Co implies that the PtAgBi NPLs serve as nucleation sites, which supports our proposed formation mechanism of PtAgBiCo NPLs. The XRD patterns show that there are no diffraction peaks of the individual metals Pt, Ag, Bi or Co that would indicate the formation of single-phase alloys. In the PtAgBi alloy, all of the diffraction peaks are shifted to higher angles toward Bi (Fig. S5a†) compared with the standard diffraction patterns for Pt and Ag phases, while after the addition of cobalt the diffraction peaks are shifted toward Pt and Co (Fig. S5b and c†). This is indicating that the introduction of Co generates tetrametallic NPLs with only one kind of morphology (triangular) (Fig. 1a and S6†). The most important feature of the present synthesis protocol is the generation of high yield size control tetrametallic NPLs through relying on the presence of twin planes (Fig. S7†) creating favourable sites for the addition of adatoms by oxidative etching, which when coupled with Co(ii) species and the I/O_2_ pair with polyol reduction, could significantly alter the reduction kinetics and change the crystallinity of PtAgBi NPLs and thus induce the formation of PtAgBiCo triangular NPLs of equal shape (Fig. 1a). To explain the formation mechanism of PtAgBiCo NPLs, through systematic studies we observed firstly the high-aspect ratio of triangular and hexagonal PtAgBi NPLs and secondly, after the incorporation of Co, the formation of triangular PtAgBiCo NPLs resulted from different reaction times (3 h and 6 h, Fig. 1 and 2). It is clear that there are very important crystallographic considerations that derive the growth of prismatic shapes in our reaction system. Among the precursors of our reaction system, Ag has six-fold symmetry and the alloy of Ag can easily form twinned crystals.^3,24^ Along these lines, it also has been proved that the alloy of Bi can form twinned crystals^25^ (Fig. S7c†). Due to this (six-fold symmetry), the product obtained in the early stages of the reaction can be considered to have a hexagonal structure,^27^ and the six surfaces where the twin plane terminates the stacking fault of the twin causes {111}-faces^16,17^ to form alternating concave- and convex-type faces designated “A-type” and “B-type”, respectively (Fig. S8a†). The addition of atoms to the B-type face is unfavourable as an adatom attaching to surface has limited stabilization energy due to the presence of only three nearest atomic neighbors; comparatively, the A-type edges are more favourable for adatom deposition due to the presence of four nearest neighbors^17^ (Fig. S8a and b†). These A-type faces (concave) are often referred to as reentrant grooves and serve as primary site for atomic addition, facilitating the transformation of hexagonal NPLs into triangular NPLs.^26^ To confirm the formation mechanism and disclose the details, a series of control experiments were conducted. It turns out that after 20 min reaction time, triangular prisms start to generate, and as the reaction time increases further, more triangular prisms are generated (Fig. S9b and c†). After one hour of reaction time, most of the products obtained are triangular prisms which act as reentrant grooves (Fig. S9d†) for hexagonal and triangular NPLs.^27–29^

The products of PtAgBi alloy obtained after 3 and 6 hours contained hexagonal and triangular NPLs (Fig. S9f and g†). The presence of different sizes and types of NPLs are due to many factors, and of these, one reason is the supply of precursors which have been consumed.^17^ The second reason for the presence of different sized hexagonal NPLs Fig. 2e and f is the different numbers of stacking faults defined by the particles at the time of twinning.^27^ The third reason for the formation of different sized hexagonal and triangular NPLs (Fig. 2a) is the development of plates from the seeds having different numbers of twin planes at the seed level^17^ (Fig. S7c†). An even number (DT = double twin) of twin planes generates hexagonal NPLs and an odd number (ST = single twin) of twin planes produces triangular NPLs.^27^ The addition of Co among Pt, Ag and Bi changes the crystallinity of the seeds and the reduction kinetics^9^ to provide more seeds to reentrant grooves (Fig. S10b)† and forms triangular NPLs after 3 and 6 hours reaction time (Fig. S10d and e†) compared to PtAgBi NPLs after the same reaction time, which is schematically shown in Fig. S11.† To make sure of the existence of the twin planes, there is still no quantitative knowledge or parameters to get control over the twin structure formed during the nucleation process,^14^ so we only rely on the introduction of an oxidative etchant (Fe(ii)/Fe(iii) or Cu(I)/Cu(ii)) to selectively etch away twins and thus we obtained single crystalline nanostructures (Fig. S12†) instead of NPLs. Keeping in mind this crystal symmetry of the starting nuclei and internal structure of the PtAgBi alloy, we introduced cobalt in our reaction system with the increase of the reaction time. Surprisingly, we observed that the product obtained after 6 h reaction time has selectively >90% triangular NPLs (Fig. 1a). We thus propose that, in our reaction system, one of the metal precursors is silver along with an I^–^-ligand, and O_2_ is present throughout the entire reaction. As well as this, most of the nuclei and seeds are twinned, which generates PtAgBi hexagonal and triangular NPLs, while the incorporation of Co alters the internal structure of the PtAgBi alloy (double twin into single twin) along with providing seeds required to generate just triangular NPLs having nearly an equal size.

In order to prove our hypothesis, we carried out a systematic study and first examined the role of O_2_. The reaction solutions were bubbled with Ar gas for one hour to get rid of oxygen and after the reaction time of 3 and 6 hours for the PtAgBi and PtAgBiCo NPLs, it is observed that the yield of NPLs drops dramatically (Fig. S13†), suggesting the important role of O_2_, which is in line with Zhang and co-workers.^12^ Further, amongst the halogens, iodide appears to be the most suitable reagent for NPLs formation, and in Fig. S14a† we examined its role carefully. In the absence of iodide ions no NPLs can be obtained and it discovered that a trace amount (60 mg) of iodide plays a determining role in directing the NPLs growth (Fig. S15c†). It also turns out that the crystallinity alteration depends on the type of foreign cation (Co^2+^, Cu^2+^) used in the synthesis. We thus believe that the incorporation of Co can substantially alter the surface formation energies and changes the crystallinity into a single twin, while Cu^2+^ changes the crystallinity into a single crystalline product.^3,30^ Therefore, for the formation of PtAgBiCo NPLs, we carefully added the Co which tightly controls the reduction kinetics^31^ of Pt, Ag and Bi, and thus obtained the high yield of triangular NPLs that is in line with Xia and co-workers.^11^ We have confirmed that the morphology of the final product is strongly dependent on the concentration of Co, and in the absence of Co, the sample mainly consists of two kind of shapes: hexagonal and triangular NPLs (Fig. 1c and d). As the concentration of Co increases to 0.005 and 0.01 millimoles, the product contains >50% and >90% triangular NPLs (Fig. S6b and c†) while nanoplates get aggregated when the concentration of Co increases further (Fig. S6d†) which confirms the kinetic control of Co over NPLs, a similar conclusion to Yin and co-workers.^9^ Thanks to oxidative etching, after the nucleation step, the nanoplates grew up as more precursor seeds were added to the surface seeds. Thus, the incorporation of Co not only altered the crystallinity through selective binding on the {111}-facets but also facilitated other nuclei through chemical etching for the formation of triangular nanoplates (Fig. S10†). The role of Co seems to be similar to that of H_2_O_2_, I^3–^ and Fe(iii)/O_2_, which facilitate the formation of Ag, Au and Pd nanoplates in high yield.^10–12^ Furthermore, this was verified by XRD and XPS; the shifting of the diffraction peaks towards higher angles clearly reveals the incorporation of Co (Fig. S5b and c†). Similarly, the XPS spectra (Fig. S16†) clearly indicate that the seeds which are required to reentrant grooves for the generation of triangular NPLs are provided by the Pt, Ag and Bi after the addition of Co. This supports our hypothesis that the incorporation of Co not only changes the reduction kinetics of the precursors but also changes the surface formation energy to generate the required Pt^0^ and Bi^3+^ seeds, while due to oxidative etching, the Ag^0^ concentration falls down below the deduction limit of XPS (Fig. S16b2†).

In order to check the role of metal precursors, we conducted additional experiments: without Ag we obtained many types of PtBi structures *e.g.* triangle, truncated triangle, diamond, ribbon, hexagon and so on^32^ (Fig. S17†). Without Bi and Co, we obtained PtAg nanosheets and PtCo nanoparticles respectively (Fig. S18†) The role of Co in the formation of triangular NPLs was also examined and we observed that the co-existence of Pt, Ag, Bi among Co is necessary (Fig. S19†). Furthermore, we replaced Pt with other noble metals (Rh, Pd, Fig. S20†) and found out the standard reaction temperature (140 °C) for multimetallic NPLs (Fig. S21†). From all the cases mentioned above, it is clear that appropriate compositions and concentrations are important for tri- and tetrametallic NPLs.

To explain the formation mechanism and catalytic role of multimetallic NPLs, the surface composition, electronic structure and interactions between Pt, Ag, Bi and Co atoms in PtAgBi and PtAgBiCo nanoplates were investigated by X-ray photoelectron spectroscopy (XPS). The Pt 4f signal of the PtAgBi NPLs (Fig. S16a1†) is deconvoluted into two components, the low energy band (Pt 4f_7/2_) at 70.61 and 71.21 eV and a high energy band (Pt 4f_5/2_) at 73.83 and 74.58 eV respectively, indicating that Pt is present in two different oxidation states, Pt(0) and Pt(ii). The peak area between Pt(0) and Pt(ii) suggests that Pt is predominately in the metallic state in PtAgBi NPLs. The Pt 4f spectrum of PtAgBiCo NPLs consists of two peaks in metallic form (Pt 4f_7/2_) at 70.48 eV and (Pt 4f_5/2_) 73.71 eV (Fig. S16b1†). The shifting of the Pt 4f signals in PtAgBiCo NPLs compared with that in PtAgBi NPLs is implying charge transfer from Co to Pt.^33^ The XPS spectrum of Ag 3d (Fig. S16a2†) in PtAgBi shows that there are two peaks at 367.2 and 373.1 eV respectively. In the presence of oxygen, the surface Ag atoms are more susceptible to oxidation^3^ in the PtAgBiCo NPLs, which verifies that the sample is free of Ag within the detection limit of the XPS technique (Fig. S16b2†), which is confirmation of silver oxidative etching from PtAgBiCo. The Bi 4f spectrum of PtAgBi NPLs can also be deconvoluted into two pairs of peaks at 156.8 eV and 158.2 eV (4f_7/2_) and at 162.2 and 163.4 eV (4f_5/2_), respectively (Fig. S16a3†). The binding energy values at 156.8 eV and 162.2 eV are assigned to pure metallic Bi(0) while the 4f_7/2_ peaks at 158.2 eV and 163.4 eV might be attributed to the Bi(iii) oxidation state on the surface of the PtAgBi NPLs.^32^ Notably, the larger proportion of Bi(iii) on the surface of PtAgBiCo NPLs (Fig. S16b3†) compared to that on the surface of PtAgBi NPLs could be attributed to alloy formation, due to the incorporation of Co amongst Pt, Ag and Bi. This indicates that the introduction of cobalt significantly changes the surface structure of PtAgBi NPLs.^25^ Similarly, the Co 2p signal of PtAgBiCo NPLs is deconvoluted into four different components. The binding energies of the peaks located at 779.30 eV and 782.5 eV (Fig. S16b4†) can be assigned to Co(0) and Co(ii) species, respectively.^34,35^ In general, the addition of a 3d transition metal (Co) to Pt-based alloy NPLs can lead to a change of the electronic structure of the alloy due to lattice strain and charge transfer.

To explore the catalytic properties of NPLs in a fuel cell cathode, an electrochemical dealloying process was adopted that can significantly improve the catalytic performance of multimetallic alloys.^20^ Prior to the test, the as-prepared PtAgBi and PtAgBiCo NPLs were treated with acetic acid for 24 hours. Pt is less likely to dissolve away by acid treatment^25^ but a portion of Bi, Ag and Co can be leached out and in the whole process of acid treatment, the size of NPLs remained unchanged. Thus, we obtained Pt-enriched porous PtAgBiCo NPLs for the ORR. In order to make sure, we analysed the NPLs through HRTEM, XRD, EDX, ICP-OES and XPS after the acid treatment. We concluded that the final product is enriched in Pt metal and the contents of the other metals, Ag, Bi and Co, decrease to some extent after the acetic acid treatment. TEM, HRTEM and line-scanning analysis (Fig. S22†) clearly reveals the effect of the acid treatment on the final product. The chemical structure of the acid-treated NPLs investigated by XRD analysis (Fig. S23†) indicates that the phase of NPLs did not change while the broadening and angle shifts of the PtAgBiCo NPLs peaks after acid treatment toward the Pt standard diffraction pattern distinctly indicates that the composition of the metals changes after the acid treatment, which also can be observed by the line scanning profile of PtAgBiCo NPLs (Fig. S24c†). We further made sure of this by EDX (Fig. S25†) and ICP-OES (Table S1†). The XPS spectra of all the products after acid treatment are shown in Fig. S26b1–b4.† Corresponding to the XRD data, the XPS data show similar trends before and after acid treatment. The spectra of Pt 4f, Ag 3d and Co 2p are shown in Fig. S26† and their oxidation states are very similar before and after acid-treatment in PtAgBiCo NPLs. However, the spectra of Bi 4f changes after acid treatment (Fig. S26b3†). Thus, it is evident that acid-treatment removes the high oxidation state of Bi from the surfaces of PtAgBiCo NPLs, which is in line with Hou’s work.^25^ ICP-OES analysis for the PtAgBi NPLs shows that the molar ratios decrease from 51.43/10.57/38 to 62.75/7.63/29.62 and for the PtAgBiCo NPLs, the molar ratios decrease from 58/5.7/24/12.30 to 67.33/4/18.67/10 which also confirms the partial removal of Ag, Bi, and Co metals from the alloy after the acetic acid treatment.

**Fig. 3:**
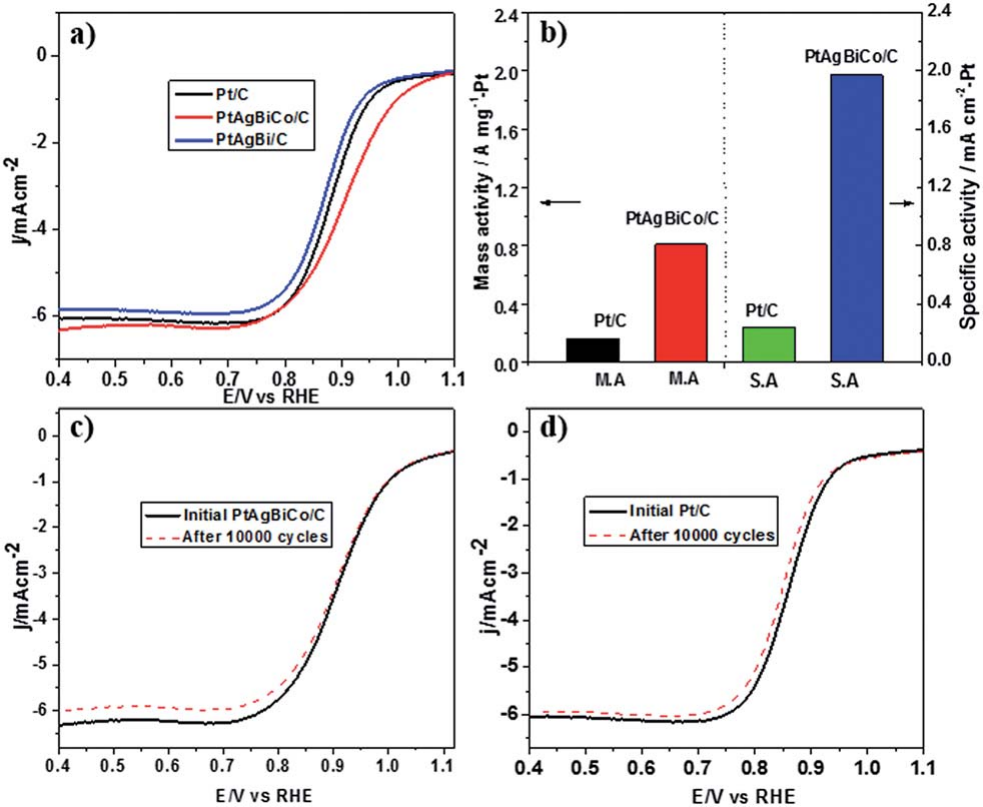
(a) ORR polarization curves for Pt/C catalyst, PtAgBi/C catalyst and PtAgBiCo/C catalyst, and (b) comparison of mass activities and specific activities of Pt and PtAgBiCo/C catalysts at 0.9 V. (c and d) ORR polarization curves for PtAgBiCo/C and Pt/C catalysts before and after 10 000 potential cycles in O_2_-saturated 0.1 M HClO_4_ solution from 0.6 to 1.0 V *vs.* RHE.

Prior to the electrochemical tests, the samples were supported on carbon black. The electrochemical performance of the PtAgBi/C and PtAgBiCo/C catalysts for the oxygen reduction reaction were first investigated using a rotating disk electrode, and compared with a state-of-the-art commercial Pt/C catalyst (JM, 20 wt% Pt). Fig. 3A illustrates the ORR polarization curves of PtAgBi/C, PtAgBiCo/C and a commercial Pt/C catalyst in O_2_-saturated 0.1 M HClO_4_ solution at a rotation rate of 1600 rpm with a scan rate of 10 mV s^–1^. The mass activity of the PtAgBiCo/C catalyst is 0.81 A m g_Pt_^–1^ at 0.90 V which is almost 5 times higher than that of the commercial Pt/C (0.16 A m g_Pt_^–1^). The specific activity of the PtAgBiCo/C catalyst (1.95 mA cm^–2^) is also improved by 8 times compared to Pt/C (0.24 mA cm^–2^). The specific electrochemical active surface area (ECSA) of the PtAgBiCo/C catalyst, calculated by using a CO-stripping method, is 41 m^2^ g_Pt_^–1^ (Fig. S27†).

The Tafel slope value of PtAgBiCo/C is less than that of the Pt/C catalyst as shown in Fig. S28† which proves that the PtAgBiCo/C catalyst has improved kinetic behaviour and higher current density than that of Pt/C. In order to evaluate ORR durability, the ORR activities of Pt/C and PtAgBiCo/C catalysts were measured before and after 10 000 cycles of accelerated durability tests (ADTs) in oxygen saturated 0.1 M HClO_4_ solution at a scan rate of 50 mV s^–1^. After 10 000 cycles of the durability test, there is almost no shift in the ORR polarization curve for the PtAgBiCo/C catalyst (Fig. 3c), while commercial Pt/C shows an obviously negative shift in ORR polarization curves (Fig. 3d). After 10 000 sweeping cycles, the PtAgBiCo/C catalyst shows only a loss of 4.2% in mass activity, while commercial Pt/C shows the 45% loss in mass activity under the same conditions (Fig. 3b). The better ORR performance of PtAgBiCo/C catalyst could be due to the stable morphology and surface structure of the nanoplates during the ORR reaction (Fig. S29†). Meanwhile, after 30 000 ADT cycles the performance of Pt/C is drastically reduced as compared to the PtAgBiCo/C catalyst under the given conditions (Fig. S30a†). The possible reason for this drastic reduction in performance could be due to aggregation^36^ of the Pt/C catalyst.

**Fig. 4:**
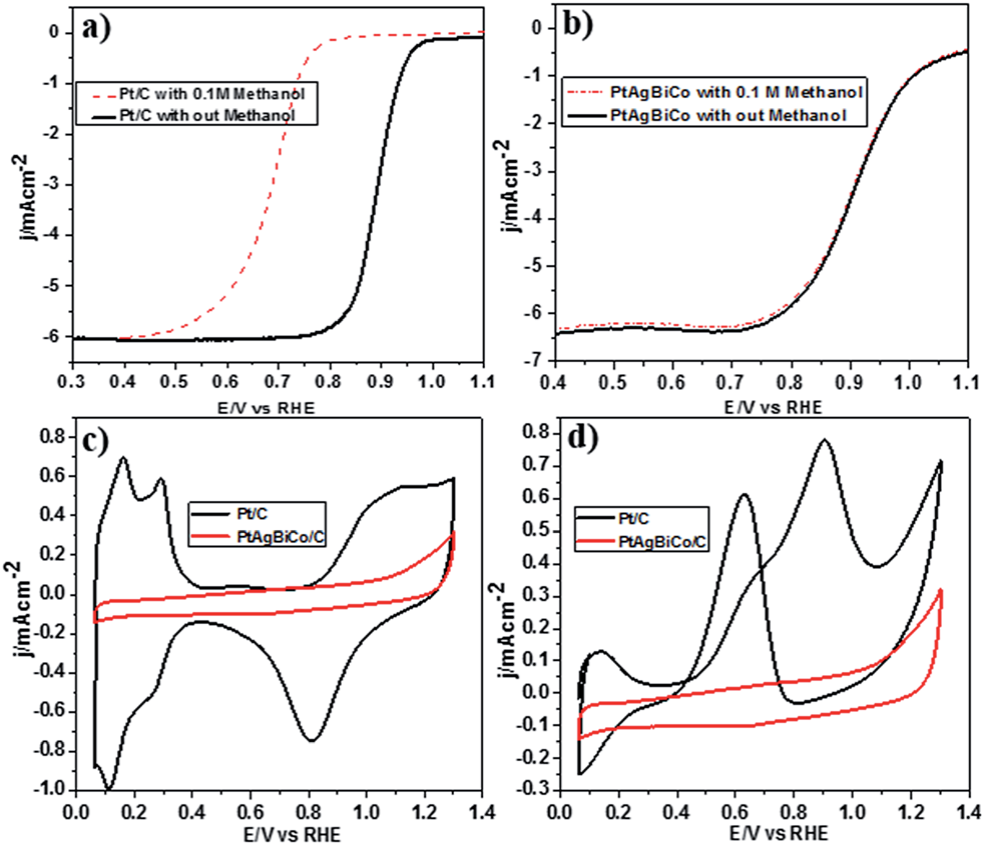
ORR polarization curves for (a) Pt/C catalyst with and without 0.1 M methanol, (b) PtAgBiCo/C catalyst in O_2_-saturated solution with and without 0.1 M HClO_4_ at a rotation rate of 1600 rpm. CV curve of Pt/C catalyst and PtAgBiCo/C catalyst in N_2_-saturated (c) 0.1 M HClO_4_, and (d) 0.1 M HClO_4_ + 0.1 M CH_3_OH.

It is well documented that the power efficiency of a fuel cell dramatically decreases because of methanol permeation to the cathode, and thus this is a significant challenge in DMFCs. To investigate the methanol tolerance of the PtAgBiCo/C catalyst, we performed ORR tests in oxygen-saturated 0.1 M HClO_4_ solutions that contained 0 and 0.1 M methanol at a rotation rate of 1600 rpm. Fig. 4 shows the ORR polarization curves of the PtAgBiCo/C and Pt/C catalysts in O_2_-saturated 0.1 M HClO_4_ solution with 0.1 M methanol solution and without methanol solution. It is evident that the half wave potential of the commercial Pt/C catalyst undergoes a dramatic negative shift in the presence of methanol (Fig. 4a). As for the catalyst, the half wave potential shows almost no degradation and the current density also remains unchanged compared with that in the methanol-free solution (Fig. 4b). Thus the as-prepared PtAgBiCo/C electrocatalyst avoided the methanol poisoning caused by methanol cross-over in a DMFC that causes an unwanted current drop. As a contrast, in the presence of methanol, the decreased activity of commercial Pt/C for the ORR is due to the higher catalytic activity towards methanol oxidation. For the commercial Pt/C catalyst, a sharp Pt oxide peak appears without methanol at 0.9 V in the CV curve, but in the presence of methanol, methanol oxidation peaks appear instead of the Pt oxide peak (Fig. 4c and d), while there is no methanol oxidation peak in the CV curve of the PtAgBiCo/C catalyst, which demonstrates that the as-prepared catalyst has better selectivity for the ORR than for the methanol oxidation reaction. The lack of Hupd features in the CV curve of the PtAgBiCo/C catalyst (Fig. 4c) showed that the hydrogen adsorption region was greatly reduced compared to the Pt/C electrocatalyst; this behavior is associated with the presence of bismuth species, in which Bi inhibits hydrogen adsorption on platinum surfaces.^19,37^ The better performance along with superior methanol tolerance of the PtAgBiCo/C compared to Pt/C demonstrates that the porous structure of PtAgBiCo/C is favourable for the ORR because the nanoporous structure (Fig. S31†) of the catalyst offers more reaction sites and enhances the activity *via* the so-called nanoconfinement effect^20,38^ by ensuring easy transport of electrons and adsorption of oxygen from the reaction medium. In the literature it is reported that the increased distance between Pt atoms and the strain effect produces more favourable sites for electrochemical activity,^39^ while Bi metal is catalytically inert for the methanol oxidation reaction in acidic medium and at least three adjacent Pt sites in a proper geometry are necessary for methanol oxidation to occur.^40,41^ Therefore, PtBi-alloy catalysts are virtually immune to CO poisoning when treated with carbon monoxide.^19,40,42^ So this geometric effect and the distortion of the three Pt sites that are required for methanol oxidation by the presence of bismuth^19,43^ might be responsible for the superior methanol tolerance of the as-prepared PtAgBiCo/C catalyst. So it is evident that the presence of Bi prevented methanol oxidation, while the incorporation of Co not only generated the triangular NPLs by altering the crystallinity but also enhanced the catalytic activity by increasing the available band vacancies, as transition metals have more band vacancies than noble metals,^19,44,45^ which confirms our idea of alloy formation among the precursors. Moreover, the electron-transfer number (*n*) during ORR was calculated by according to the well-known Koutecky–Levich (K–L) equation^46^ (Fig. S32†). The obtained value of *n* was 3.98 for the PtAgBiCo/C catalyst. This result is in close agreement with previous studies of O_2_ reduction on Pt based catalysts.^47^

To make sure that the Bi alloy hindered methanol oxidation, we synthesized PtAg, PtBi and PtCo nanostructures respectively under the given conditions (Fig. S18†) and observed that only the PtBi alloy exhibits methanol tolerance and there is no methanol oxidation peaks in the CV curve of the PtBi catalyst (Fig. S33c2†), while the methanol oxidation peak appears in the CV curve of the PtAg/C and PtCo/C catalysts (Fig. S33a2 and b2†). So PtAgBiCo NPLs can be used as a new class of high performance electrocatalyst for the development of DMFCs with tolerance to methanol poisoning.

## Conclusions

In summary, multimetallic nanoplates have been synthesized by a two step method. It is believed that the introduction of cobalt plays a very important role in the alteration of crystallinity to obtain a high morphological yield of triangular nanoplates and to improve the catalytic activity of the alloy, because the presence of the transition metal may increase the number of band vacancies and thereby increase the catalytic activity. The possible introduction of Bi into the alloy might also increase the methanol tolerance. Porous tetrametallic PtAgBCo nanoplates exhibit superior catalytic performance and long-term catalytic durability for the ORR compared with the ternary PtAgBi alloy and commercial Pt/C catalysts, as well as good resistance to methanol cross-over effects, suggesting that this material is a promising candidate for use as a cathode catalyst in direct methanol fuel cells. We believe that due to the high activity and superior durability of this new material, compared to commercial Pt/C, the material would also be attractive for use in hydrogen fuel cells, and not only methanol fuel cells.
