# Supplementary material for: Shape controlled synthesis of porous tetrametallic PtAgBiCo nanoplates as highly active and methanol-tolerant electrocatalyst for oxygen reduction reaction
Source: Chem Sci. 2017 Mar 22;8(6):4292–8. doi: 10.1039/c7sc00318h (PMC5468992; doi:10.1039/c7sc00318h)
Supplement: Supplementary file 1 [file SC-008-C7SC00318H-s001.pdf]

Electronic Supplementary Material (ESI) for Chemical Science.
This journal is © The Royal Society of Chemistry 2017

Electronic Supplementary Material (ESI) for Chemical Science.

Shape Controlled synthesis of porous tetrametallic PtAgBiCo nanoplates as highly active and methanol-tolerant electrocatalyst for Oxygen Reduction Reaction.

Azhar Mahmood, Nanhong Xie, Muhammad Aizaz Ud Din, Faisal Saleem, Haifeng Lin, Xun Wang*

Key Lab of Organic Optoelectronics and Molecular Engineering, Department of Chemistry, Tsinghua University, Beijing 100084, China. E-mail: wangxun@mail.tsinghua.edu.cn

Experimental Section

Regents:

$Pt(acac)_2$, $AgNO_3$, $Cu(acac)_2$, $Fe(acac)_2$, $Pd(acac)_2$, $RhCl_3$, $Co(acac)_2$ poly(vinylpyrrolidone) (PVP; Mw 8000), were purchased from Alfa Aesar Co.Ltd, and $Bi(NO_3)_2$, KI, KBr, KCl, tris (hydroxymethyl) aminomethane, HCHO solution (37 wt%), formamide, and acetone were purchased from Sinopharm Chemical Reagent Co. Ltd . All the chemicals were analytical grade and used as received without any purification for the Synthesis of PtAgBi, PtAgBiCo nanoplates and PtAg, PtBi, PtCo, PdAgBi, RhAgBi nanostructures.

Synthesis:

Synthesis of PtAgBi Nanoplates: PtAgBi NPLs synthesis were carried out by two-step method. In the first step a mixture of 50 mg tris and 200 mg PVP was dissolved in 2 mL HCHO solution and transferred to Teflon-lined stainless steel autoclave which was heated at 200°C for 3 hours. A gel like material was obtained, after the washing and centrifugation in acetone. Then in the second step a homogeneous solution of 0.01mmol $Pt(acac)_2$, 0.01mmol $AgNO_3$, 0.01mmol $BiNO_3$, and 0.06 g KI was prepared in 2 ml formamide solvent and pour into 12 ml Teflon-lined stainless steel autoclave along with gel like material which was prepared in the first step, and kept the autoclave in oven at 140°C for 3 h. Final product was obtained after washing by using acetone.

Synthesis of* PtAgBiCo *Nanoplates: The synthesis procedure and experimental conditions for PtAgBiCo nanoplates were same as the PtAgBi nanoplates, except that, we added $Co(acac)_2$ precursor in the second step for tetrametallic nanoplates and the reaction time was increased from three hours to six hours.

Synthesis of PdAgBi and RhAgBi Nanostructures: The synthesis procedure and experimental conditions for PdAgBi and RhAgBi nanostructures were same as the PtAgBi nanoplates, except that, we replaced $Pt(acac)_2$ by $Pd(acac)_2$ for the synthesis of PdAgBi nanostructures and for RhAgBi nanostructures we replaced $Pt(acac)_2$ by $RhCl_3$ respectively.

Synthesis of PtAg, PtBi, PtCo Nanostructures: For PtAg nanostructures, synthesis were carried out by two-step method. Firstly, a mixture of 50 mg tris and 200 mg PVP was dissolved in 2 mL HCHO solution and transferred to Teflon-lined stainless steel autoclave which was heated at 200°C for 3 hours. A gel like material was obtained, after the washing and centrifugation in acetone. Secondly, a homogeneous solution of 0.01mmol $Pt(acac)_2$, 0.01mmol $AgNO_3$, and 0.06 g KI was prepared in 2 ml formamide solvent and pour into 12 ml Teflon-lined stainless steel autoclave along with gel like material which was prepared in the first step, and kept the autoclave in oven at 140°C for 3 h. Final product was obtained after washing by using acetone. Similarly, PtBi and PtCo nanostructures were synthesized, for

PtBi all the reaction conditions were similar as PtAg except we replace $AgNO_3$ by BiNO3, and for PtCo nanostructures synthesis we added $Co(acac)_2$ along with $Pt(acac)_2$.

Electrochemical Measurements:

Oxidation reduction reaction (ORR) electrochemical measurements of PtAgBi and PtAgBiCo catalyst were performed using a standard RDE system. Electrochemical test was performed on Princeton P4000 electrochemical workstation, using three electrode cell. The reported potentials are specified relative to the RHE scale. Prior to the experiment, glassy electrode was polished with alumina suspensions powder to yield a mirror finish on a felt polishing pad. Then, as prepared PtAgBi, PtAgBiCo nanoplates or Pt/C NPs were dispersed in 0.80 mL of H_2O, 0.20 mL of ethanol, and 10 µL of Nafion solution (0.05 wt%) by soniocation for more than 1 h to form a homogeneous catalyst ink. The catalyst ink was transferred to the surface of the glassy carbon (GC) electrode and dried at room temperature. ORR measurements were performed oxygen saturated 0.1 M $HClO_4$ solutions using GC-RDE at a scan rate of 10 mV s^{-1} with or without methanol at a rotation speed of 1600 rpm and CV measurements were performed in N_2-saturated solutions. The mass activity, which is the current per unit mass (molar conc. determined through ICP-OES analysis) of the Pt/C and PtAgBiCo/C Catalyst, is the corresponding current at the potential of 0.9 V RHE and mass of samples can be calculated. For CO-stripping experiment to calculate ECSA, the catalyst was saturated with high purity CO by bubbling CO into the electrolyte for 25 min. By flowing N2-gas for 15 min, the residual CO was then removed. The CO-stripping curve was recorded from 0.05 to 1.55 V with a scanning rate of 50 mV s^{-1}. The CO-ECSA value was calculated by integration of charges between 0.82 and 1.1 V using the charge between the first and second cycles. The measured charge was then normalized using the theoretical value of 420 µC cm^{-2}, mass of $catalyst_{Pt}$, and scanning rate, i.e., 50 mV s^{-1}. In CV curve the current densities were normalized reference to the geometric area of RDE(0.196cm^2).

Characterization:

Transmission electron Microscopy (TEM) and high-resolution transmission electron microscope (HRTEM) were recorded on a HITACHI H-7700 TEM with an accelerating voltage of 100 kV, and FEI Tecnai G2 F20 S-Twin high-resolution TEM equipped with energy dispersive spectrometer (EDS) analyses at 200 Kv. The samples for HRTEM analysis were prepared by dropping ethanol dispersion of samples onto 300-mesh molybdenum grids.

X-ray diffraction (XRD): XRD patterns were measured with a Bruker D8-advance X-ray powder diffractometer operated at 40 KV voltage and instrument run at a scan rate of 0.02 deg / s in the angle range of 5° to 90° and the wave length of the incident radiation was λ = 1.5418 Å.

X-ray photoelectron spectroscopy (XPS): XPS measurements were conducted on a scanning X-ray microprobe (Quantera SXM, ULVAC-PHI. INC) operated at 250 kV, 55 eV with monochromated Al Kα radiation.

ICP-OES: Elemental analysis of the samples was determined by inductively coupled plasma atomic emission spectrometry (ICP-AES, IRIS Intrepid Ⅱ XSP, Thermo Fisher).

Acknowledgements This work was supported by NSFC (21431003, 21521091) and China Ministry of Science and Technology under Contract of 2016YFA0202801.

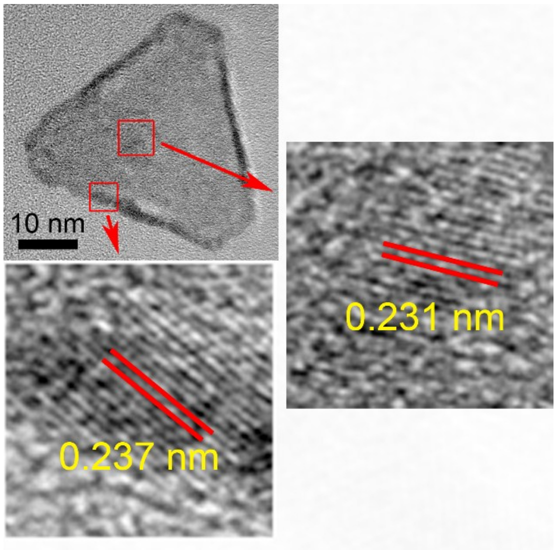

Figure S1. HRTEM image of triangular PtAgBiCo nanoplates.

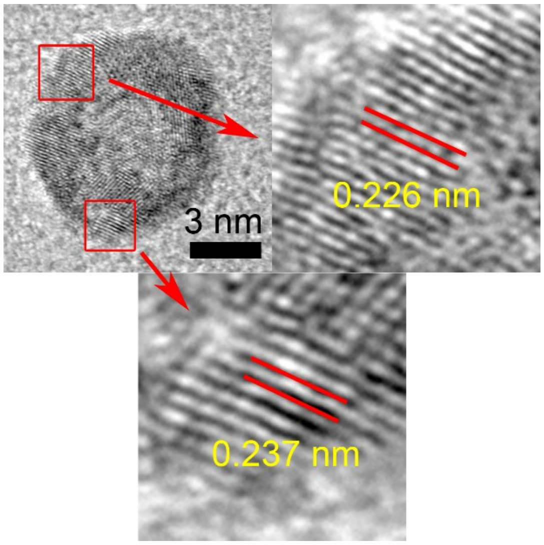

Figure S2. HRTEM image of PtAgBi nanoplates.

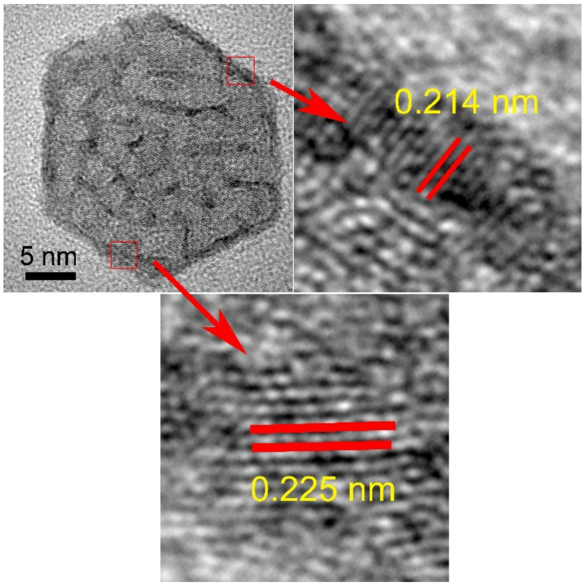

Figure S3. HRTEM image of hexagonal PtAgBi nanoplates.

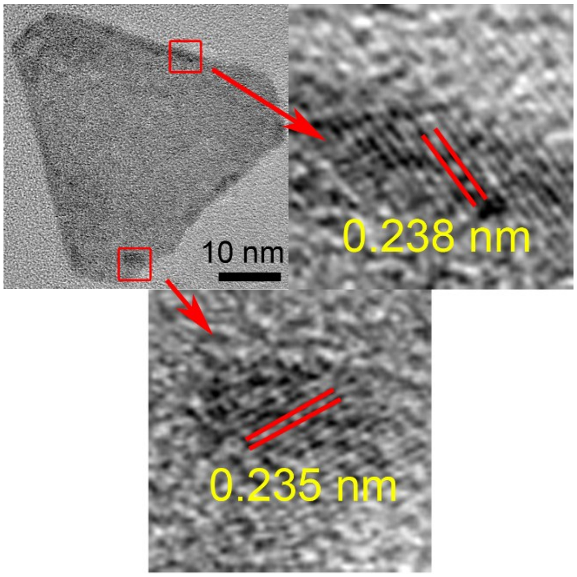

Figure S4. HRTEM image of triangular PtAgBi nanoplates.

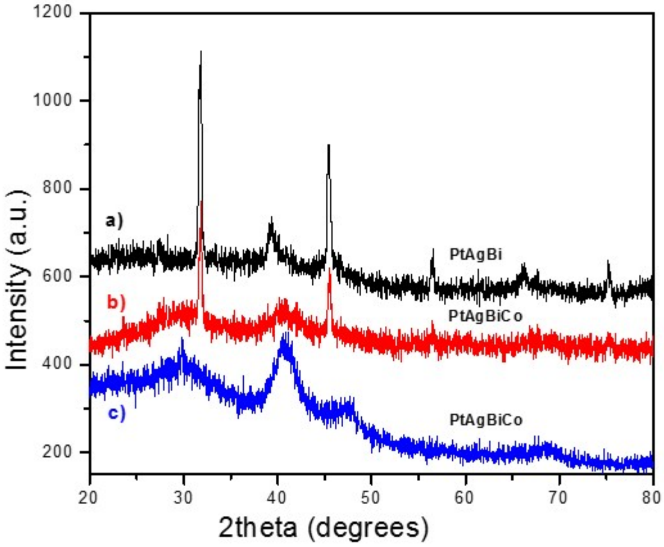

Figure S5. XRD patterns of (a) PtAgBi [1] and (b,c) PtAgBiCo [2] Nanoplates, where b) and c) are with 0.005 and 0.01 molar concentration of cobalt.

The XRD patterns shows that there are no diffraction peaks of the individual metal Pt, Ag, Bi or Co that would indicate the formation of single-phase alloys. In the PtAgBi alloy, all of the diffraction peaks are shifted to higher angles toward Bi Fig.S5a (Pt1Bi1-ICSD#58545) compared with the standard diffraction patterns for pure Pt (Pt-JCPDS no.04-0802) and Ag (Ag-JCPDS no.04-0783) references. While after addition of cobalt with low concentration, the diffraction peaks of the alloy start to shift toward Pt and Co (Co-JCPDS no.150806) references Fig.S5b. This slight shifting of the peaks after addition of low concentration of cobalt is the indication of the incorporation of cobalt and the formation of the tetrametallic PtAgBiCo alloy. Meanwhile, after the addition of an appropriate concentration of the cobalt, where we obtained tetrametallic PtAgBiCo nanoplates with only one kind of morphology (Triangular), the corresponding diffraction peaks of PtAgBiCo alloy more shifted toward Pt and Co references Fig.S5c. It is indicating that the introduction of Co, generates tetrametallic NPLs with only one kind of morphology (Triangular).

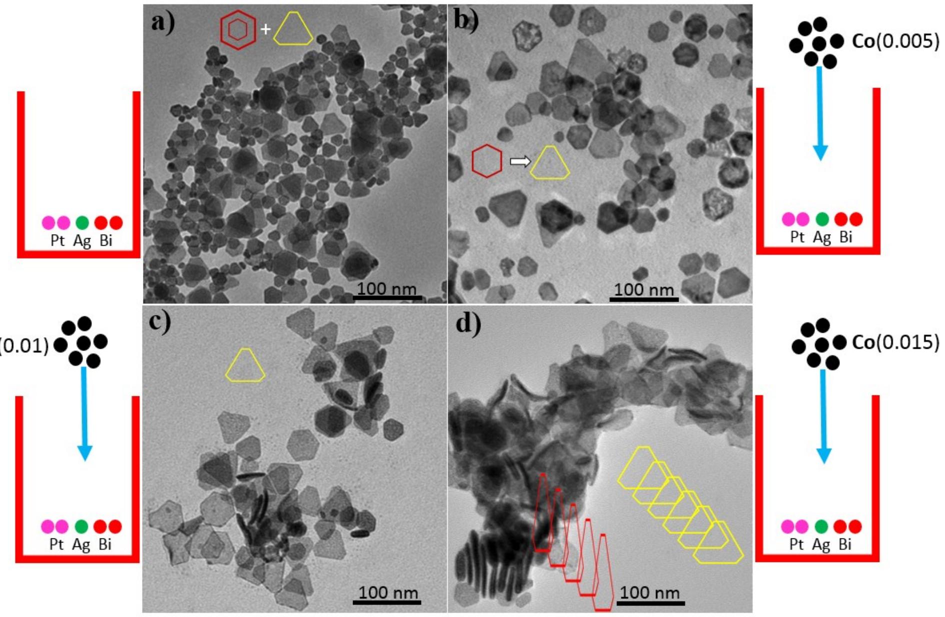

Figure S6. TEM images of nanoplates prepared with different molar ratios of $Pt(acac)_2$, $AgNO_3$, $Bi(NO3)_2$, and $Co(acac)_2$: a) 0.01:0.01:0.01, b) 0.01:0.01:0.01 and 0.005 c) 0.01:0.01:0.01 and 0.01 d) 0.01:0.01:0.01 and 0.015.

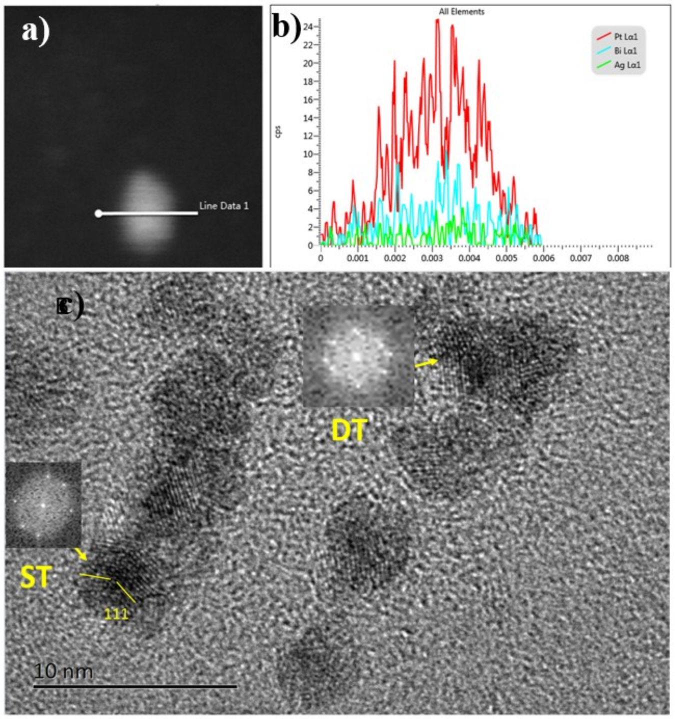

Figure S7. a) STEM-HAADF b) line-scanning profile and c) HRTEM images of PtAgBi Twin nanoparticles ST= single twin and DT= double Twin.

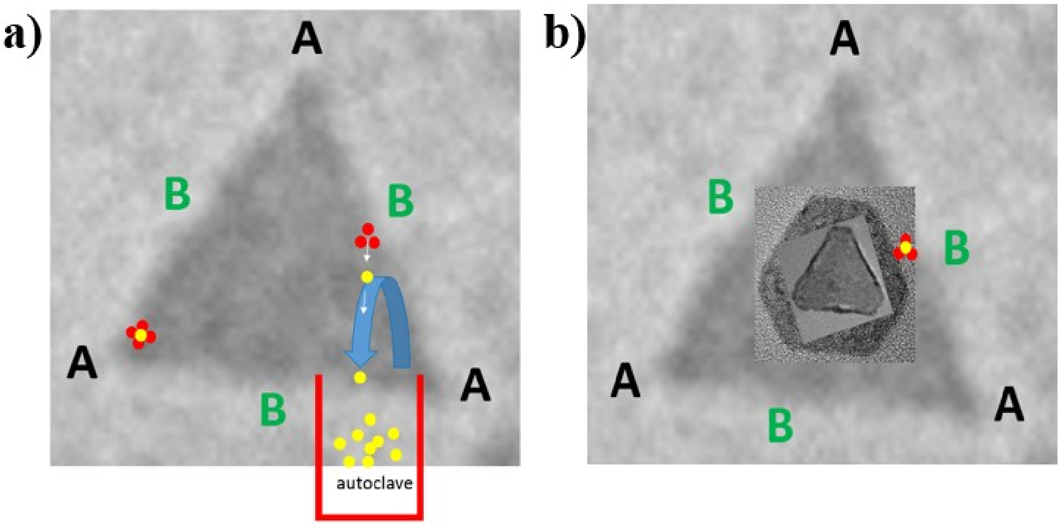

Figure S8. TEM images of as prepared PtAgBi a) reentrant groves and b) its transformation into hexagonal and triangular nanoplates.

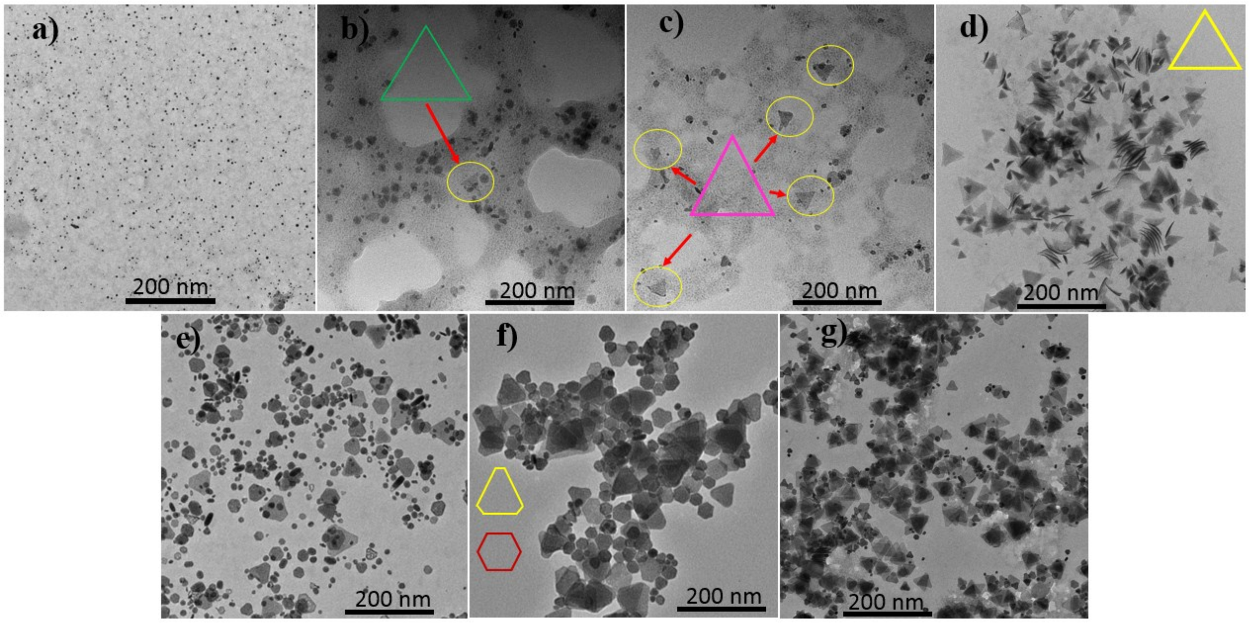

Figure S9. TEM images of as prepared PtAgBi nanostructures at a) 5 min, b) 20 min, c) 40 min, d) 60 min, e) 120 min, f) 180 min and g) 360 minutes reaction time.

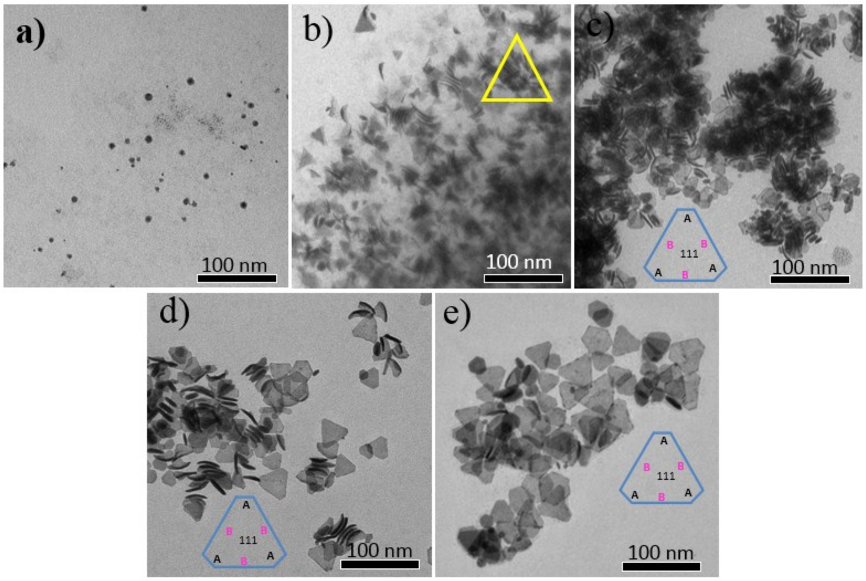

Figure S10. TEM images of as prepared PtAgBiCo nanostructures at a) 5 min, b) 60 min, c) 120 min, d) 180 min, e) 360 minutes reaction time.

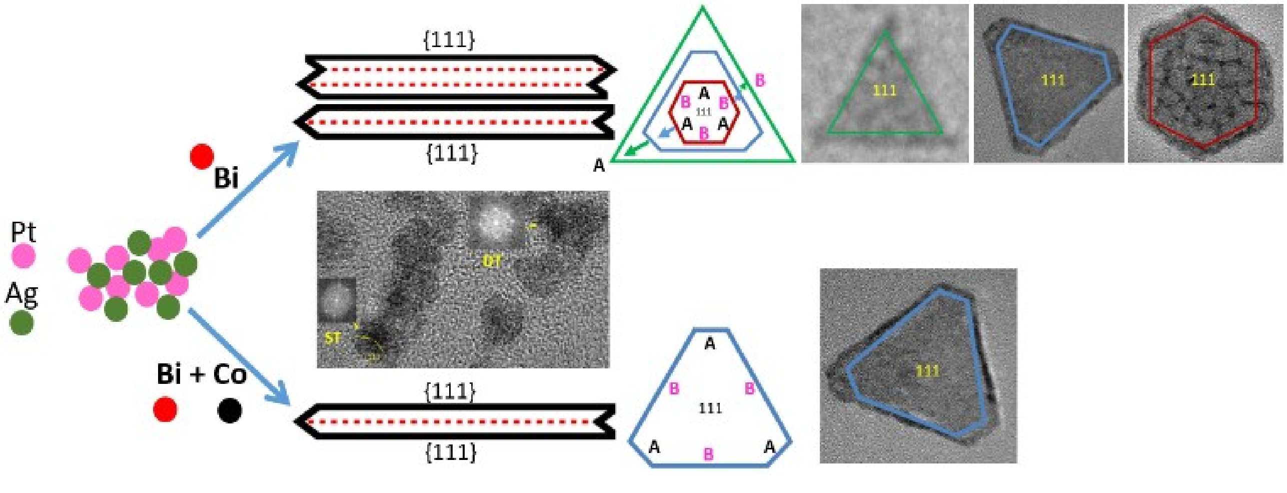

Figure S11. Schematic illustration of multimetallic nanoplates.

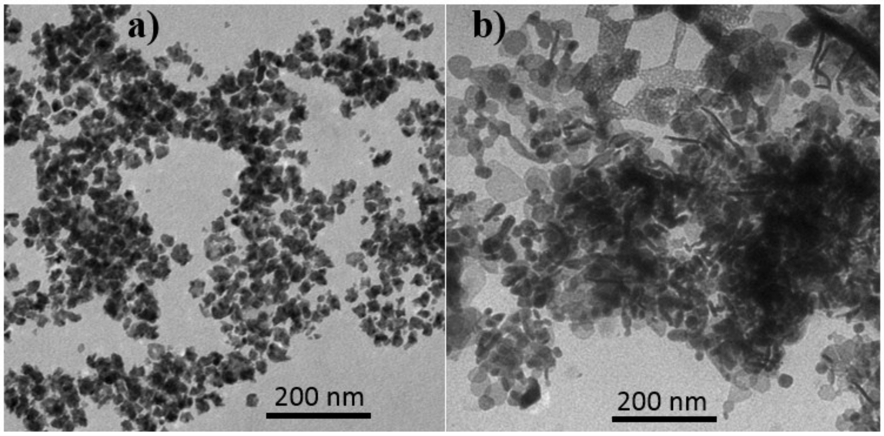

Figure S12. TEM images of as prepared a) PtAgBiFe and b) PtAgBiCu nanostructures at 180 minutes reaction time.

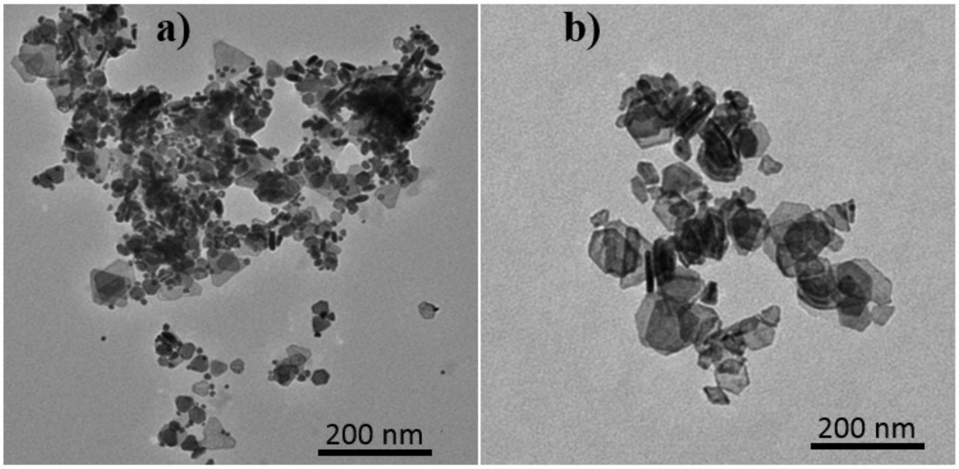

Figure S13. TEM images of as prepared a) PtAgBi nanoplates at 180 min and b) PtAgBiCo nanoplates at 360 minutes reaction time after Purged with Ar gas.

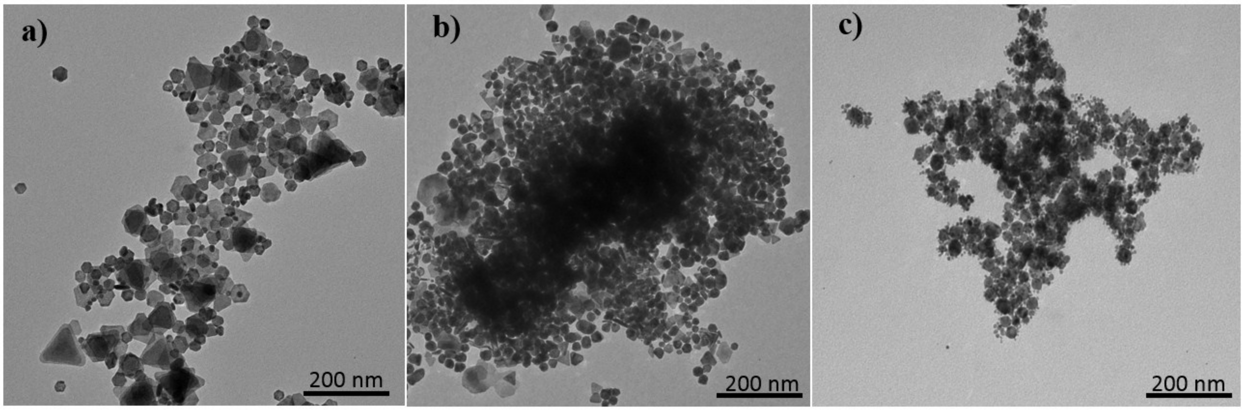

Figure S14. TEM images of the PtAgBi products collected after three hour reactions time where different halide anions were used a) KI, b) KCl, and c) KBr all the other reaction conditions were same.

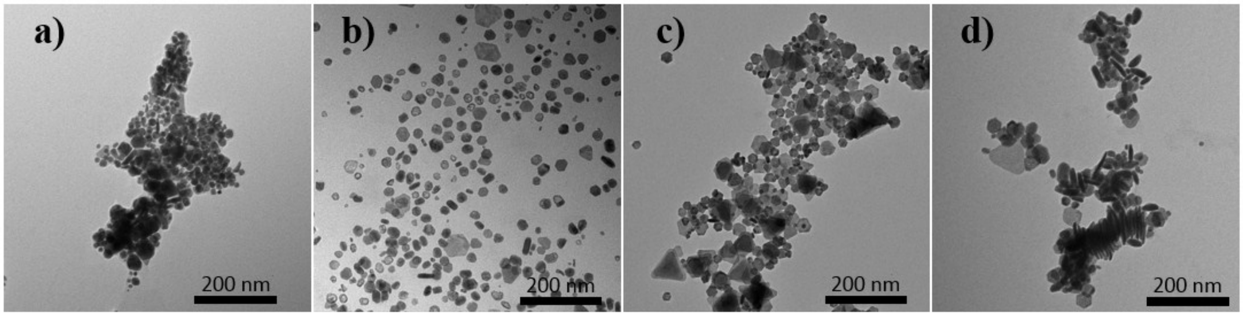

Figure S15. TEM images of as prepared PtAgBi nanoplates when KI conc is different a) 0.0 mg, b) 30 mg, c) 60 mg and d) 90 mg.

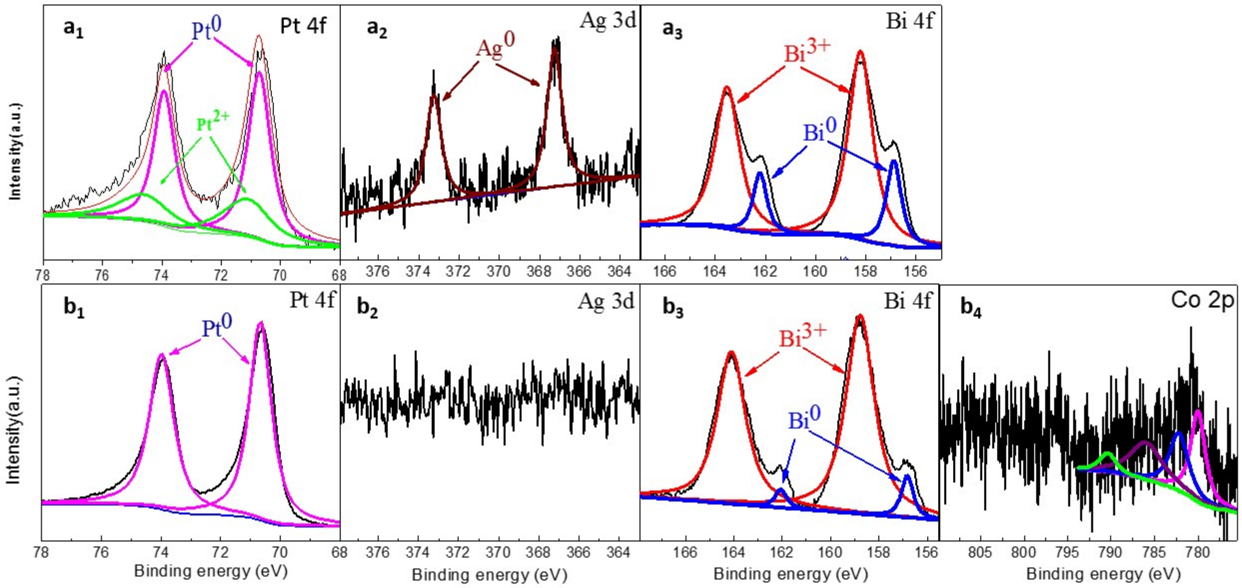

Figure S16. X-ray photoelectron spectroscopy of PtAgBi (a1, a2 and a3) and PtAgBiCo (b1, b2 b3 and b4) nanoplates.

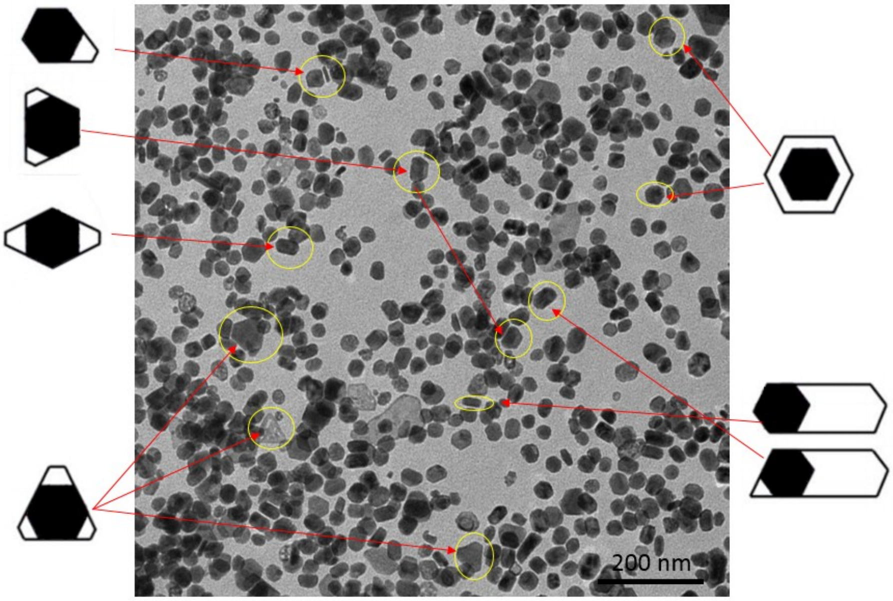

Figure S17. TEM image of PtBi alloy with different counters (Triangle, Truncated triangle, diamond, ribbons, prisms and hexagon).

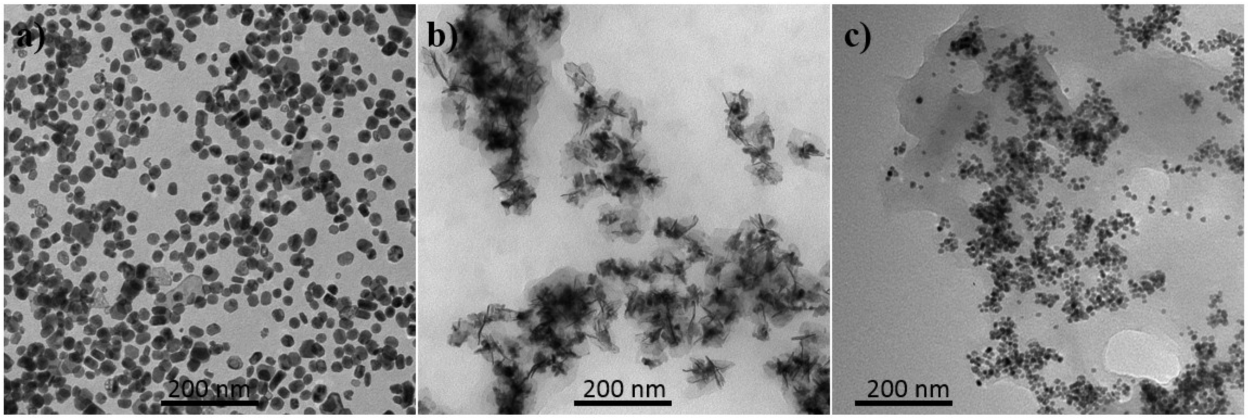

Figure S18. TEM images of different nanostructures a) PtBi, b) PtAg, c) PtCo at three hour reaction time. All the products synthesized under the standard experimental condition in the absence of a) $AgNO_3$, b) $Co(acac)_2$ and c) $AgNO_3$. Firstly, we obtained a) PtBi counters. Secondly, we conducted the experiment in the absence of bismuth, we obtained ultrathin nanosheets which we already have been reported[3].Third we obtained c) PtCo nanoparticles, also in the absence of bismuth.

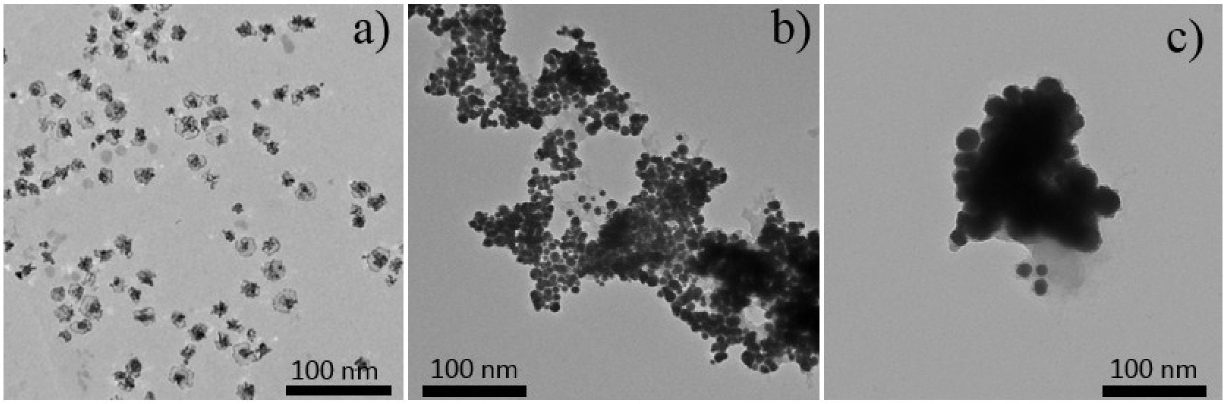

Figure S19. TEM images of different nanostructures a) PtAgCo, b) PtBiCo, c) AgBiCo at six hour reaction. All the products synthesized under the standard experimental condition in the absence of a) $BiNO_3$, b) $AgNO_3$ and c) $Pt(acac)_2$ and we obtained a) PtAgCo nanosheets, b) PtBiCo nanaopartcles, c) AgBiCo nanostructures.

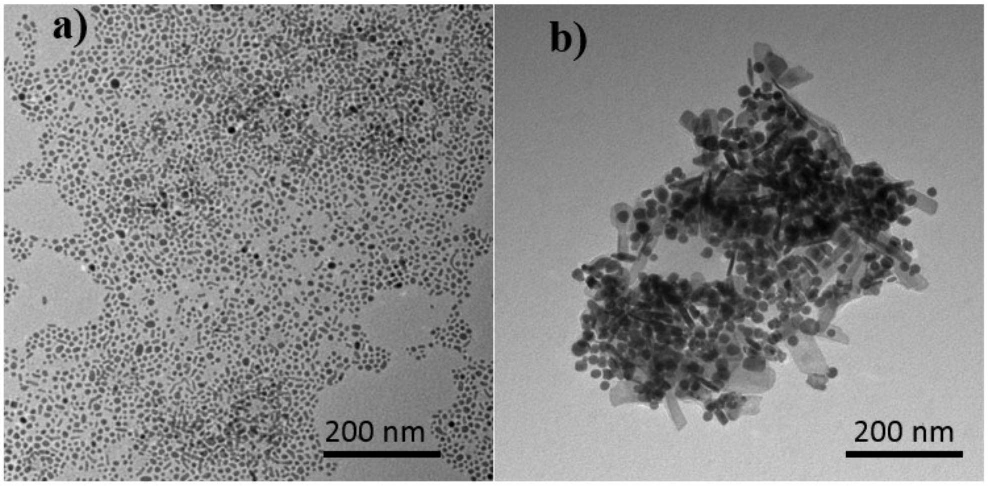

Figure S20. TEM images of as prepared a) PdAgBi and b) RhAgBi nanostructures.

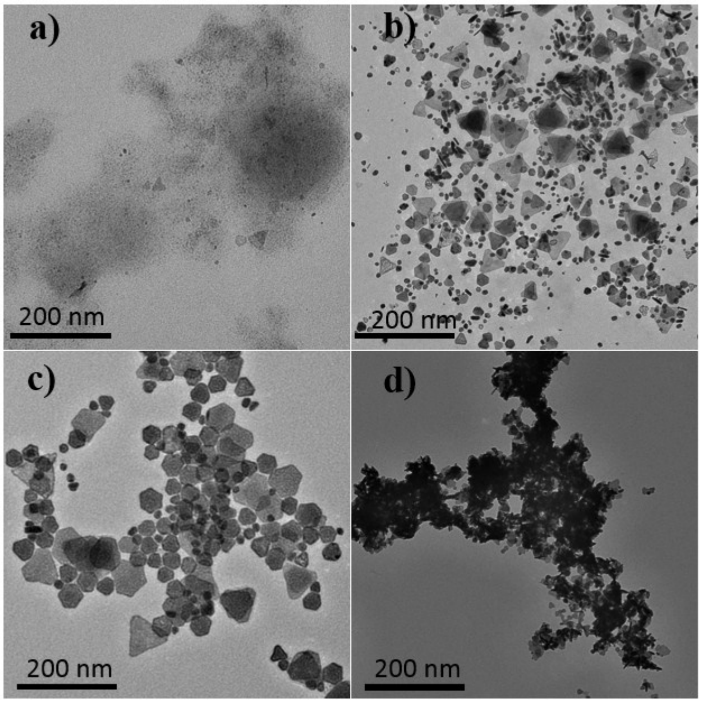

Figure S21. TEM images of PtAgBi nanostructures prepared at different reaction temperature a) 100 ^{O}C, b) 120 ^{O}C, C) 140 ^{O}C and 170 ^{O}C.

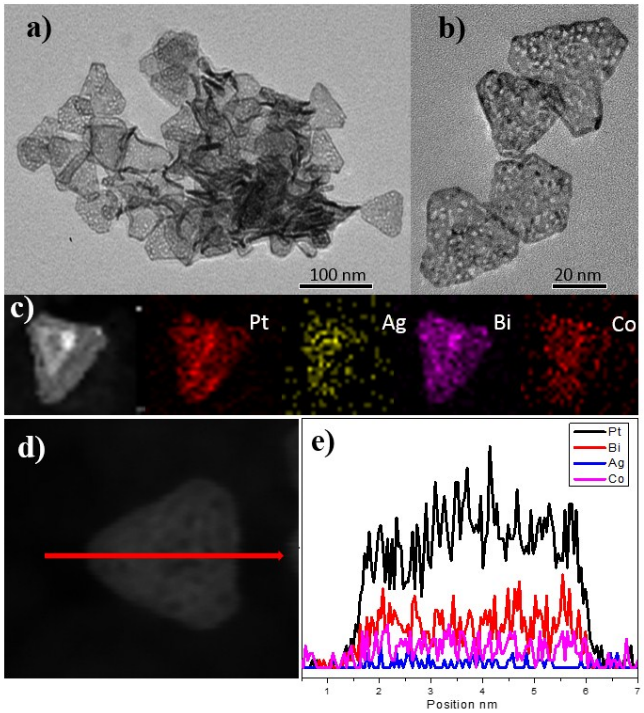

Figure S22. a) TEM, b) HRTEM c) EDX mapping d) STEM-HAADF images and d) Line scanning profile of PtAgBiCo nanoplates after acetic acid treatment.

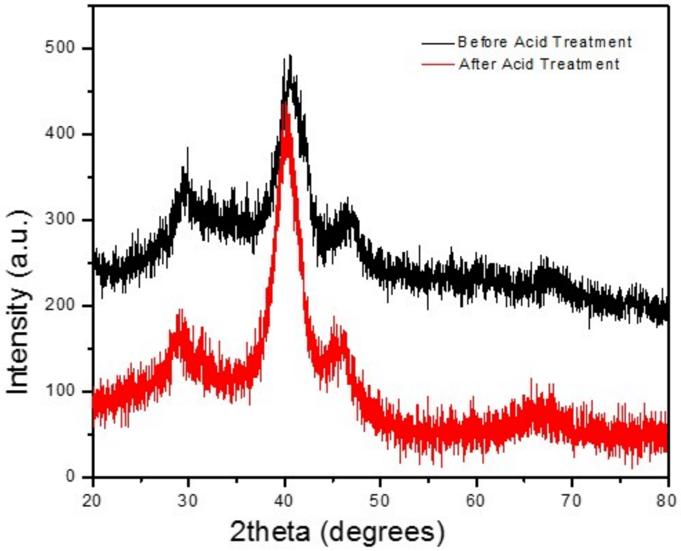

Figure S23. XRD patterns of PtAgBiCo nanoplates before and after acetic acid treatment.

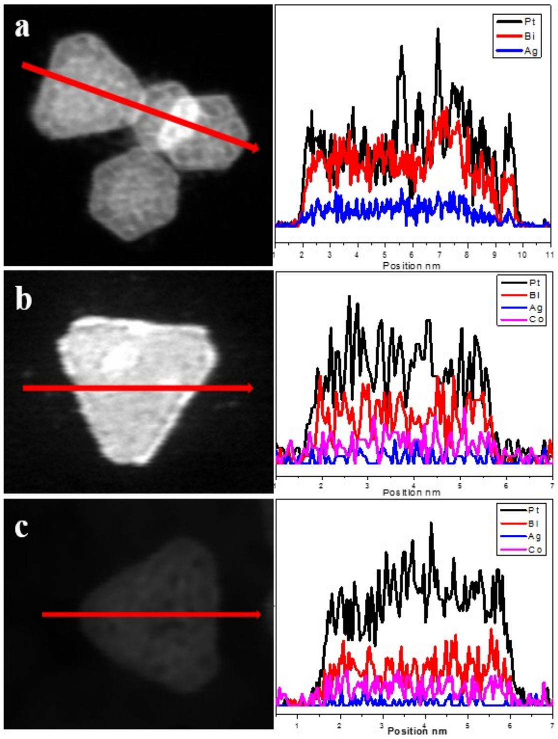

Figure S24. Line scanning profiles before acid treatment a) PtAgBi, b) PtAgBiCo and after acetic acid treatment c) PtAgBiCo.

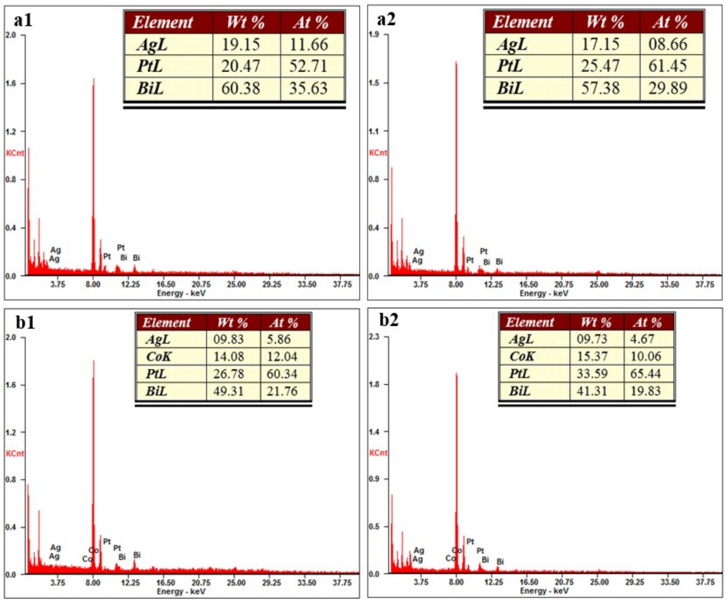

Figure S25. A representative EDX spectrums of nanoplates before acid treatment a1) PtAgBi, b1) PtAgBiCo and after acetic acid treatment a2) PtAgBi, b2) PtAgBiCo.

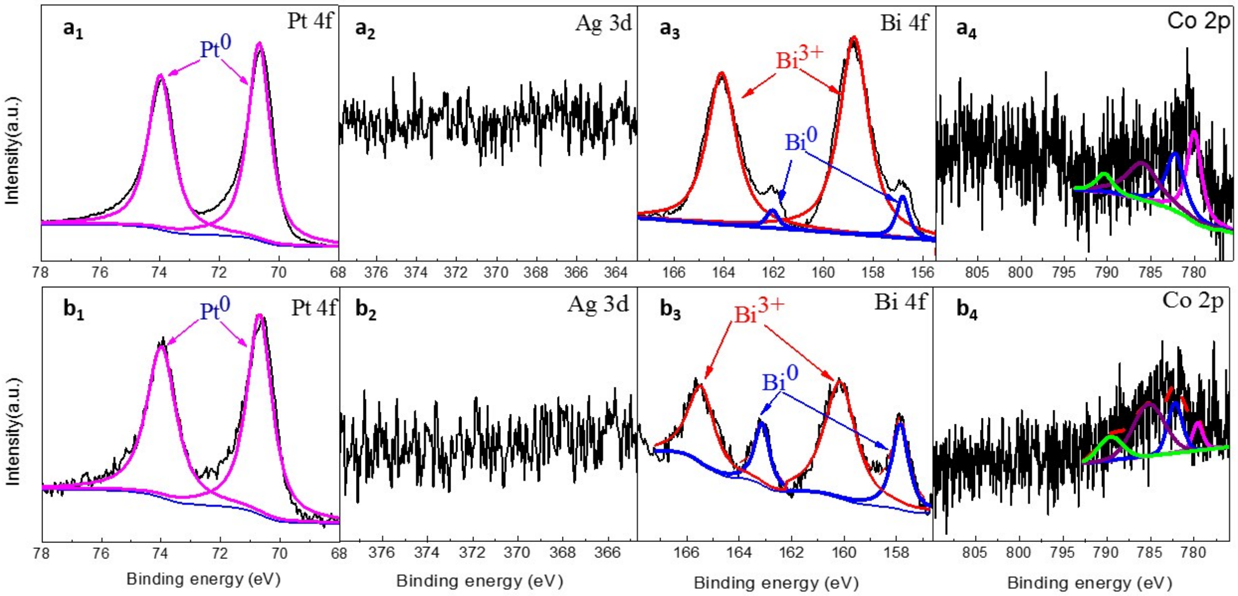

Figure S26. X-ray photoelectron spectroscopy of PtAgBiCo nanoplates before acid treatment (a1, a2, a3 and a4) and after acetic acid treatment (b1, b2 b3 and b4).

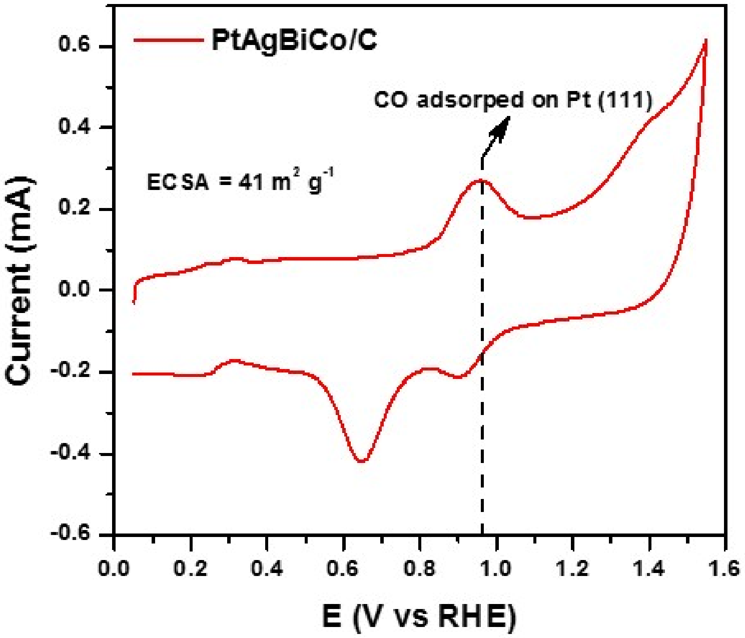

Figure S27. CV curve of PtAgBiCo/C catalyst by CO-Stripping method.

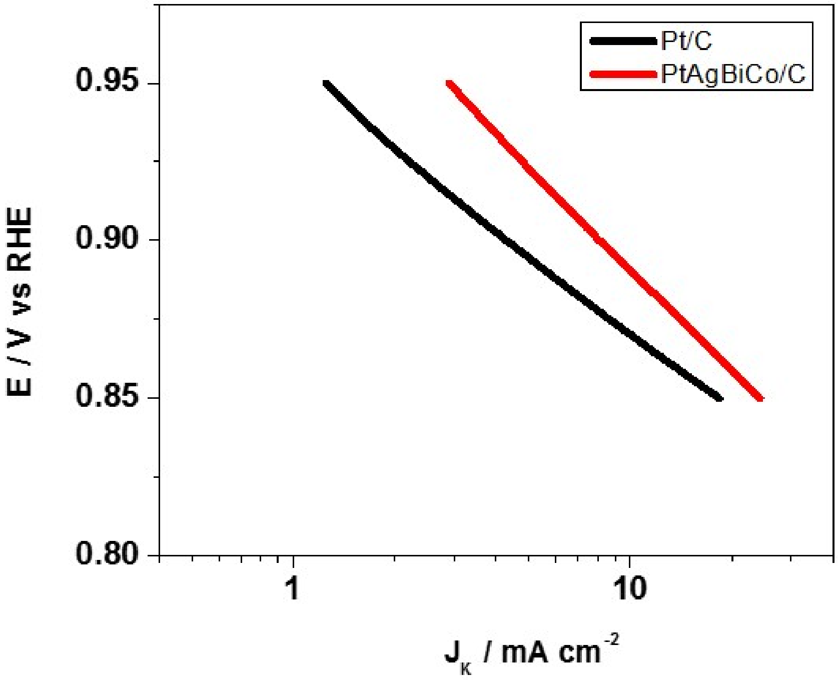

Figure S28. Tafel plot of Pt/C and PtCuBiMn/C at a rotation speed of 1600rpm in 0.1 $HClO_4$ Solution with a sweep rate of 50mV/s

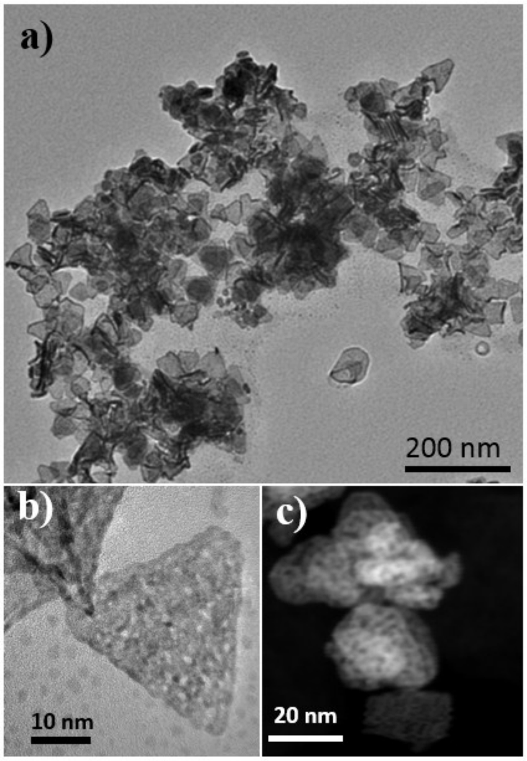

Figure S29. a) TEM, b) HRTEM and c) STEM images of the PtAgBiCo/C nanoplates. The images show that the morphology and surface structure of the nanoplates sample are largely maintained after the ORR reaction, which can be responsible for the excellent performance.

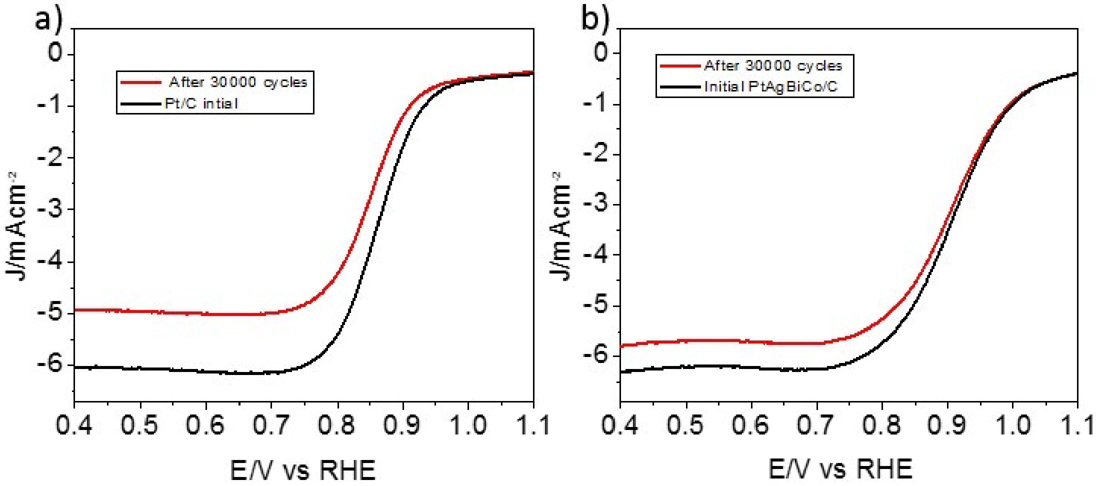

Figure S30. ORR polarization curves a) Pt/C and b) PtAgBiCo/C catalysts before and after 30000 potential cycles in oxygen 0.1 M HClO4 solution from 0.6 to 1.0 V vs. RHE at a scan rate of 50 mV s^{-1}.

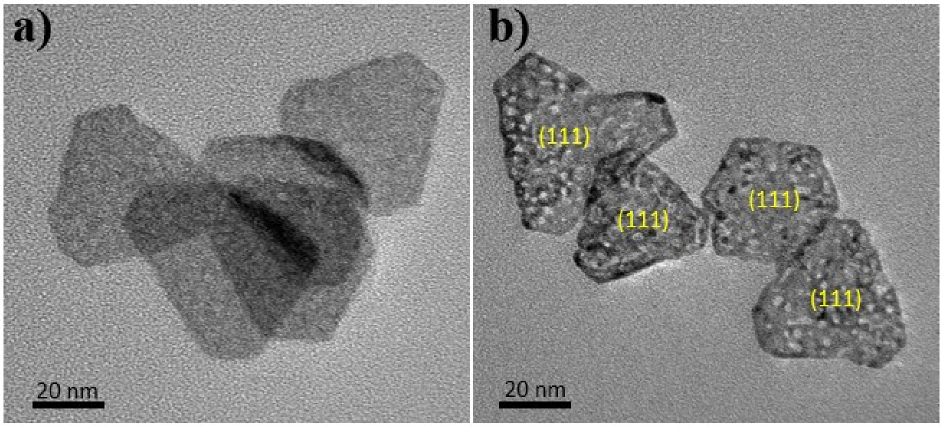

Figure S31. Porous structures of as prepared PtAgBiCo nanoplates a) TEM and b) HRTEM images.

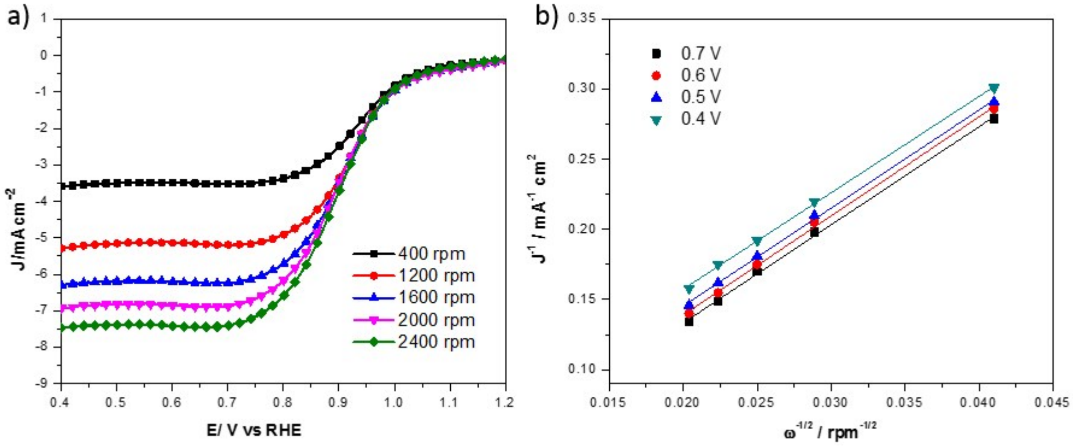

Figure S32. a) RDE voltammograms at different rotating speeds for PtAgBiCo/C, b) Koutecky−Levich plots of PtAgBiCo/C in O_2 saturated 0.1 M $HClO_4$ solution from 0.6 to 1.0 V vs. RHE.

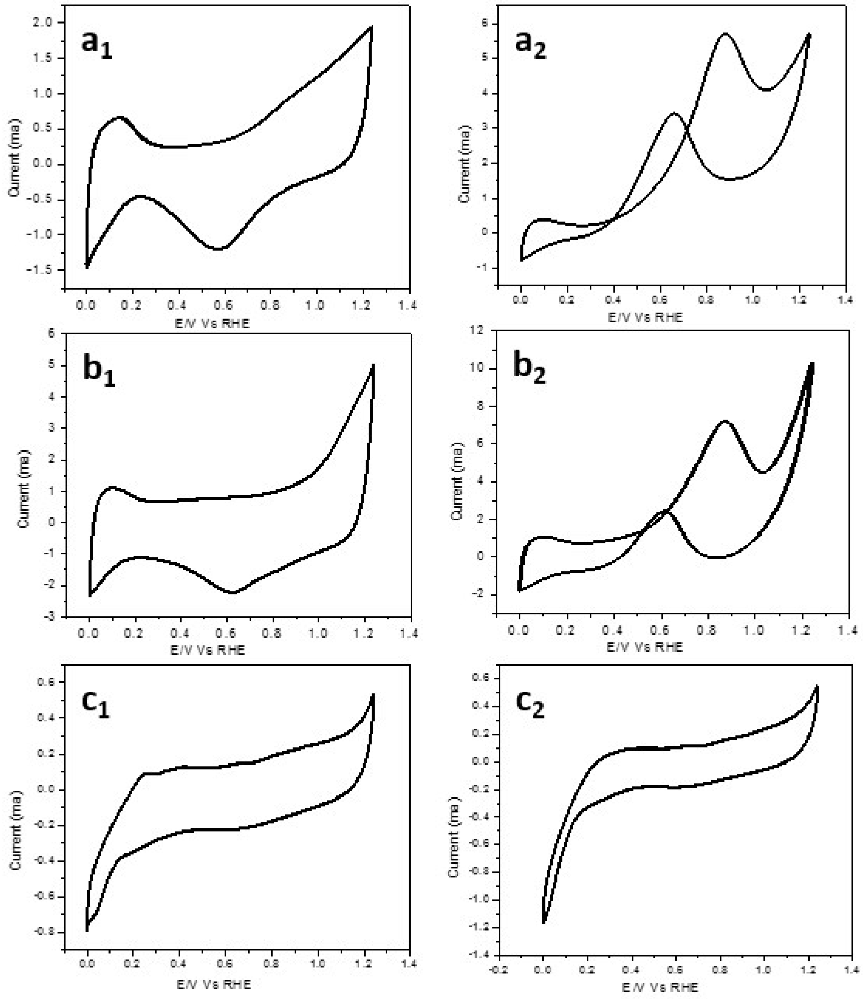

Figure S33. CV curve of PtAg catalyst in N_2-saturated (a1) 0.1M $HClO_4$ (a2) 0.1 M $HClO_4$+0.1M CH_3OH. CV curve of PtCo catalyst in N_2-saturated (b1) 0.1M $HClO_4$ (b2) 0.1 M $HClO_4$+0.1M CH_3OH, and CV curve of PtBi catalyst in N_2-saturated (c1) 0.1M $HClO_4$ (c2) 0.1 M $HClO_4$+0.1M CH_3OH.

Sample	Mole ratio determined by ICP-OES
Pt-Ag-Bi Nanoplates	51.43:10.57:38
Pt-Ag-Bi after acetic acid treatment	62.75:7.63:29.62
Pt-Ag-Bi-Co Nanoplates	58:5.7:24:12.30
Pt-Ag-Bi-Co after acetic acid treatment	67.33:4:18.67:10

Table S1. The composition of PtAgBi and PtAgBiCo nanoplates determined by ICP-OES, before and after acetic acid treatment.

	Mass activity of Pt-Ag-Bi-Co/C Catalyst Amg_{Pt}^{-1}		
Sr.No	MA	Average	Standard Deviation **σ**
1	0.81 A $mgpt^{-1}$	0.812 A $mgpt^{-1}$	0.0346
2	0.81 A $mgpt^{-1}$		
3	0.816 A $mgpt^{-1}$		

Table S2. Mass Activity of PtAgBiCo/C Catalyst.

References:

1 A. Remona, K. Phani, *Fuel Cells* 2011, **11**, 385-393.

2 S. I. Lim, M. Varón, I. Ojea-Jiménez, J. Arbiol and V. Puntes, *Chemistry of Materials*, 2010, **22**, 4495-4504.

3 A. Mahmood, F. Saleem, H. Lin, B. Ni, X. Wang, *Chem.Commun* 2016, **52**, 10547-10550.
